# Mechanism of Action of Thalassospiramides, A New Class of Calpain Inhibitors

**DOI:** 10.1038/srep08783

**Published:** 2015-03-05

**Authors:** Liang Lu, Michael J. Meehan, Shuo Gu, Zhilong Chen, Weipeng Zhang, Gen Zhang, Lingli Liu, Xuhui Huang, Pieter C. Dorrestein, Ying Xu, Bradley S. Moore, Pei-Yuan Qian

**Affiliations:** 1 KAUST Global Collaborative Research, Division of Life Science, School of Science, Hong Kong University of Science and Technology, Clear Water Bay, Hong Kong, China; 2 Skaggs School of Pharmacy & Pharmaceutical Sciences, University of California at San Diego, La Jolla, California 92037, United States; 3 Department of Chemistry, Hong Kong University of Science and Technology, Clear Water Bay, Hong Kong, China; 4 Department of Pharmacology, University of California at San Diego, La Jolla, California 92037, United States; 5 Center for Marine Biotechnology and Biomedicine, Scripps Institution of Oceanography, University of California at San Diego, La Jolla, California 92037, United States; 6 School of Life Science, Shenzhen University, Nanhai Ave 3688, Shenzhen, Guangdong Province, 518060, China

## Abstract

Thalassospiramides comprise a large family of lipopeptide natural products produced by Thalassospira and Tistrella marine bacteria. Here we provide further evidence of their nanomolar inhibitory activity against the human calpain 1 protease. Analysis of structure-activity relationship data supported our hypothesis that the rigid 12-membered ring containing an α,β-unsaturated carbonyl moiety is the pharmacologically active functional group, in contrast to classic electrophilic “warheads” in known calpain inhibitors. Using a combination of chemical modifications, mass spectrometric techniques, site-directed mutagenesis, and molecular modeling, we show the covalent binding of thalassospiramide's α,β-unsaturated carbonyl moiety to the thiol group of calpain's catalytic Cys115 residue by a Michael 1,4-addition reaction. As nanomolar calpain inhibitors with promising selectivity and low toxicity from natural sources are rare, we consider thalassospiramides as promising drug leads.

Calpain is a calcium-dependent cysteine protease that participates in many signal transduction events by catalyzing the proteolysis of specific peptides in target substrates^1^,^2^,^3^. Deregulation of calpain action has been identified in pathologies such as neurological disorders, muscular dystrophies, cortical cataracts, cancer, and inflammation^4^,^5^,^6^,^7^,^8^,^9^. To date, more than 200 calpain inhibitors have been reported with most being synthetic peptides and peptidomimetics that target active site residues^10^. A common feature of these inhibitors is the presence of a classical electrophilic warhead (e.g., aldehyde, α-ketocarbonyl, and epoxysuccinyl) to interact with the active site cysteine residue (Cys115) of calpain^11^,^12^,^13^. However, major hindrances in the clinical application of these classical inhibitors are their poor selectivity for calpain, tendency to interact with other cysteine proteases, and high potential for toxicity^14^,^15^,^16^.

Recently, we characterized 14 new and 2 known thalassospiramide lipopeptides from several Thalassospira and Tistrella marine bacterial species (see Fig. 1) and revealed their novel biosynthetic pathways^17^. Among these analogues, six were evaluated for their potent inhibitory activity against human calpain 1 protease (HCAN1). Although differences in bioactivity were as large as 20-fold, all tested thalassospiramides were active at nanomolar concentrations, which suggests they are thus far the most potent calpain inhibitors retrieved from natural sources^13^,^14^. Interestingly, the lack of the classical warhead and the presence of a common 12-membered ring system suggest that thalassospiramides may represent a new class of calpain inhibitors.

## Results

### Bioassay and Chemical Modifications

**Figure 2:**
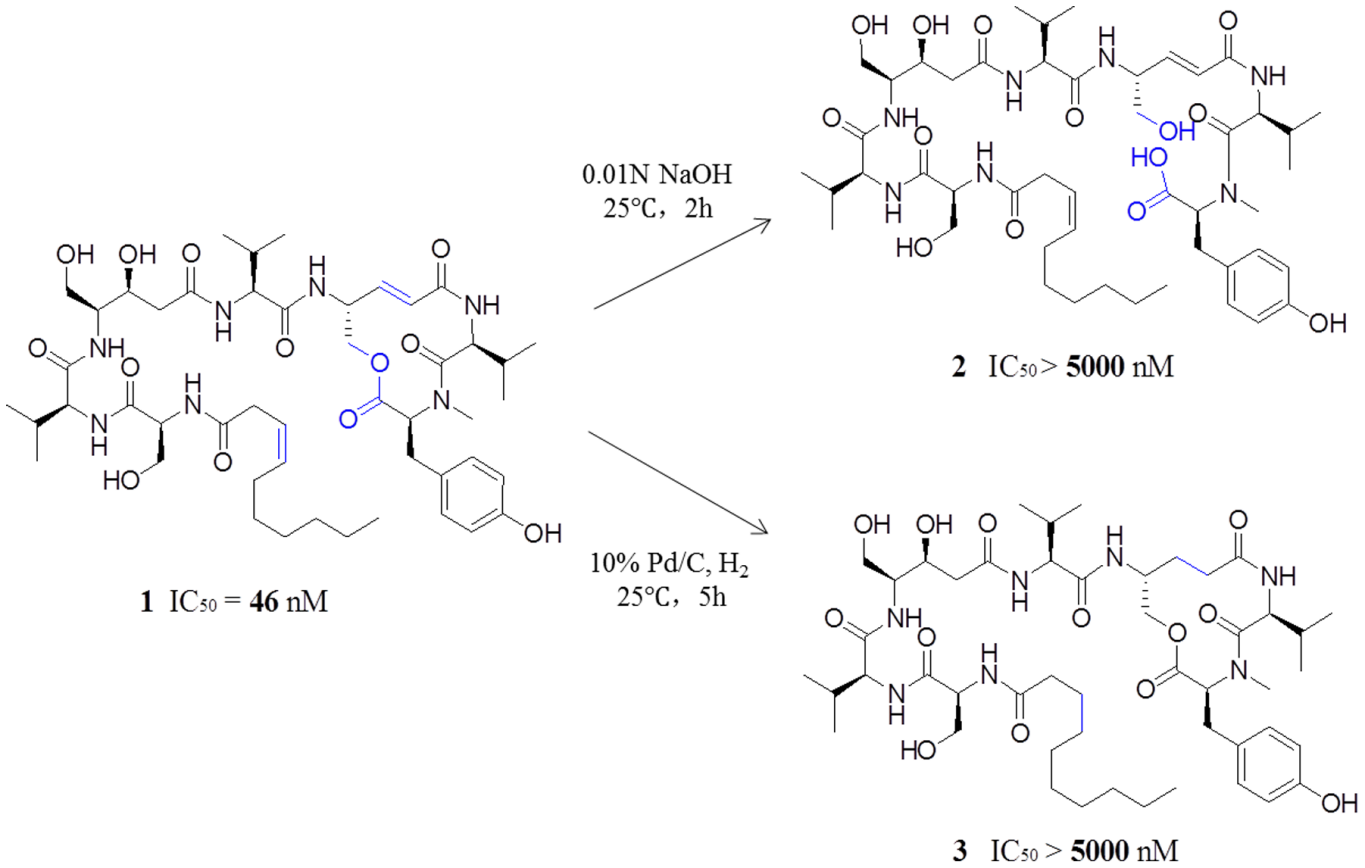
Chemical modifications of 1 and the comparison of IC_50_ values against HCAN1. Both modifications (ester hydrolysis to **2** and double-bond saturation to **3**) led to loss of bioactivity.

We accumulated all previously reported thalassospiramide analogues and evaluated their calpain 1 inhibitory activity using a fluorescence-based assay. The result showed that all thalassospiramides possessed nanomolar-level inhibitory activity against human calpain 1 (see Table 1), which suggests that the conserved 12-membered ring system with its electrophilic, unsaturated amide group might be the pharmacologically active moiety. To test this hypothesis, thalassospiramide A (**1**) was hydrolyzed at the ester position to **2** as well as hydrogenated at the double bond to **3** (see Fig. 2). In both cases, the products were 100-fold less active in the calpain inhibitory assay, strongly indicating that the intact 12-membered ring system is a critical element for the inhibitory activity. Reduction of **1** to **3** also resulted in the saturation of the acyl side chain, which, based on natural thalassospiramide analogues in the series, does not significantly influence the overall calpain bioactivity (see Table 1). These results supported our hypothesis that the α,β-unsaturated carbonyl moiety in the 12-membered ring system is essential for the inhibitory activity of calpain. We therefore predicted that Cys115 of calpain attacks the double bond of the unsaturated amide via a Michael-type 1,4-addition to form a covalent linkage between the inhibitor and protein. A similar binding mechanism was reported between the active site Thr1 residue of the 20S proteasome and the bacterial natural product syringolin A, a potent proteasome inhibitor that also contains an α,β-unsaturated amide in a 12-membered ring system^18^.

### Human Calpain 1 Sample Analysis by MALDI-TOF MS

**Figure 3:**
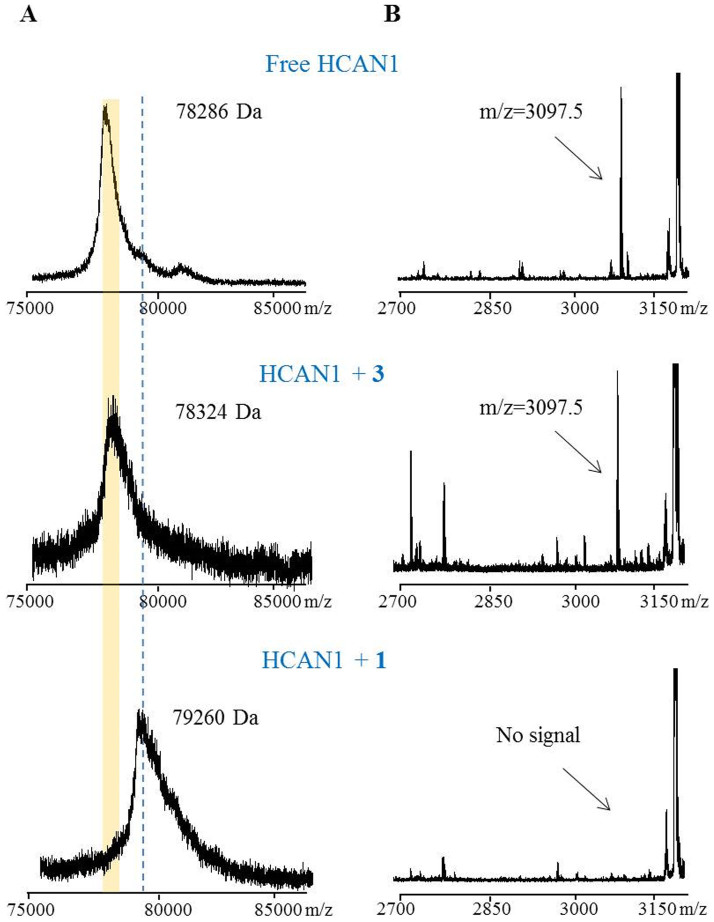
MALDI-TOF MS analysis of calpain samples. A) Intact protein analysis of free HCAN1, HCAN1 + **3** and HCAN1 + **1** samples. Compared with free HCAN1, an obvious shift could be detected in the HCAN1 + **1** sample, which has a molecular weight similar to **1** (957.5 Da). B) Analysis of the three samples after trypsin digestion. Signal of original peptide (*m/z* = 3097.5) completely disappeared in the HCAN1 + **1** sample.

Top-down and bottom-up mass spectrometry analyses were next used to explore this postulated mode of action^19^,^20^,^21^,^22^. Excess **1** and **3** were added to HCAN1 and incubated separately before being subjected to MALDI-TOF analysis. The results revealed that **1** (957.5 Da) formed a covalent adduct with calpain, as the *m/z* value was shifted by approximately 974 Da in comparison to the control sample of “free” HCAN1 (see Fig. 3A). We measured only a 1:1 (HCAN1 to **1**) complex despite using excessive amounts of **1**, suggesting a specific interaction between HCAN1 and **1**. Conversely, the HCAN1 + **3** complex did not yield a significant mass shift (see Fig. 3A), as anticipated, which is consistent with the loss of the electrophilic olefin in the 12-membered ring of **1**. These findings support the specific binding of **1** to just a single calpain amino acid residue.

To explore the nature of the covalent linkage between thalassospiramide and calpain, we digested three samples (free HCAN1, HCAN1 + **1**, and HCAN1 + **3**) with trypsin and analyzed the products by MALDI-TOF/TOF MS. The results revealed that the Cys115-containing peptide fragment TDICQGALGDC_115_WLLAAIASLTLNDTLLHR (cal. *m/z* = 3097.6) could be detected in the free HCAN1 and HCAN1 + **3** samples but not in the HCAN1 + **1** sample (see Fig. 3B and Supplementary Fig. 1) in support of the proposed mechanism. Unfortunately, using MALDI-TOF we were unable to detect the anticipated tryptic peptide fragment with **1** (*m/z* = 4055.1)^23^,^24^.

### Protein Mutation, Expression, and Identification

Our previous MALDI-TOF MS result showed that only one molecule of **1** binds per calpain (see Fig. 3A), which indicates that the interaction between calpain and thalassospiramide is highly specific. If Cys115 is the specific attachment site, then a Cys115-mutant calpain should not form a covalent bond with **1** and therefore no mass shift should be observed. To test this hypothesis, the protease core of human calpain 1 (domains I-II of μ-calpain, “μI-II” as abbreviation), which retains the functional and structural elements of intact calpain^25^, was mutated to replace the Cys115 active site residue with Ala (μI-IIC115A). Native and mutated μI-II proteins were expressed, purified and their sequences verified (see Supplementary Fig. 3, 4 and 5). However, the calculated and observed masses of the two proteins, as measured by FT-ICR-MS, were in discordance (μI-II, cal. exact mass: 42239.9 Da, obs. exact mass: 42467.9 Da; μI-IIC115A, cal. Exact mass: 42207.9 Da, obs. exact mass: 42360.1 Da). Considering that the observed mass deviations of μI-II and μI-IIC115A were 228 and 152 amu, respectively, and that both are multiples of 76, we reasoned that these differences were most likely due to the addition of β-mercaptoethanol (β-ME, 76.0 Da) from the storage buffer^26^. In order to verify that the observed mass deviations of the intact protein were actually caused by β-ME adducts, we performed a brief (30 minute) trypsin digestion of μI-II, to be followed by MS analysis, without the use of additional reducing agents such as TCEP^27^. HPLC fractionation and MS/MS analysis of the resulting peptides showed that three of the five cysteine residues were modified by β-ME (cys49, cys108 and Cys115; see Supplementary Fig. 6). This is consistent with the crystal structure of μI-II^28^ (PDB file: 1ZCM) in which only these three cysteine residues reside on the solvent-accessible surface of the protein. Verification of the formation of these β-ME adducts clearly explained the observed masses for both μI-II (+228 Da, 3x β-ME) and μI-IIC115A (+152 Da, 2x β-ME).

### Expressed Protein Sample Analysis by FT-ICR and Q-TOF MS

**Figure 4:**
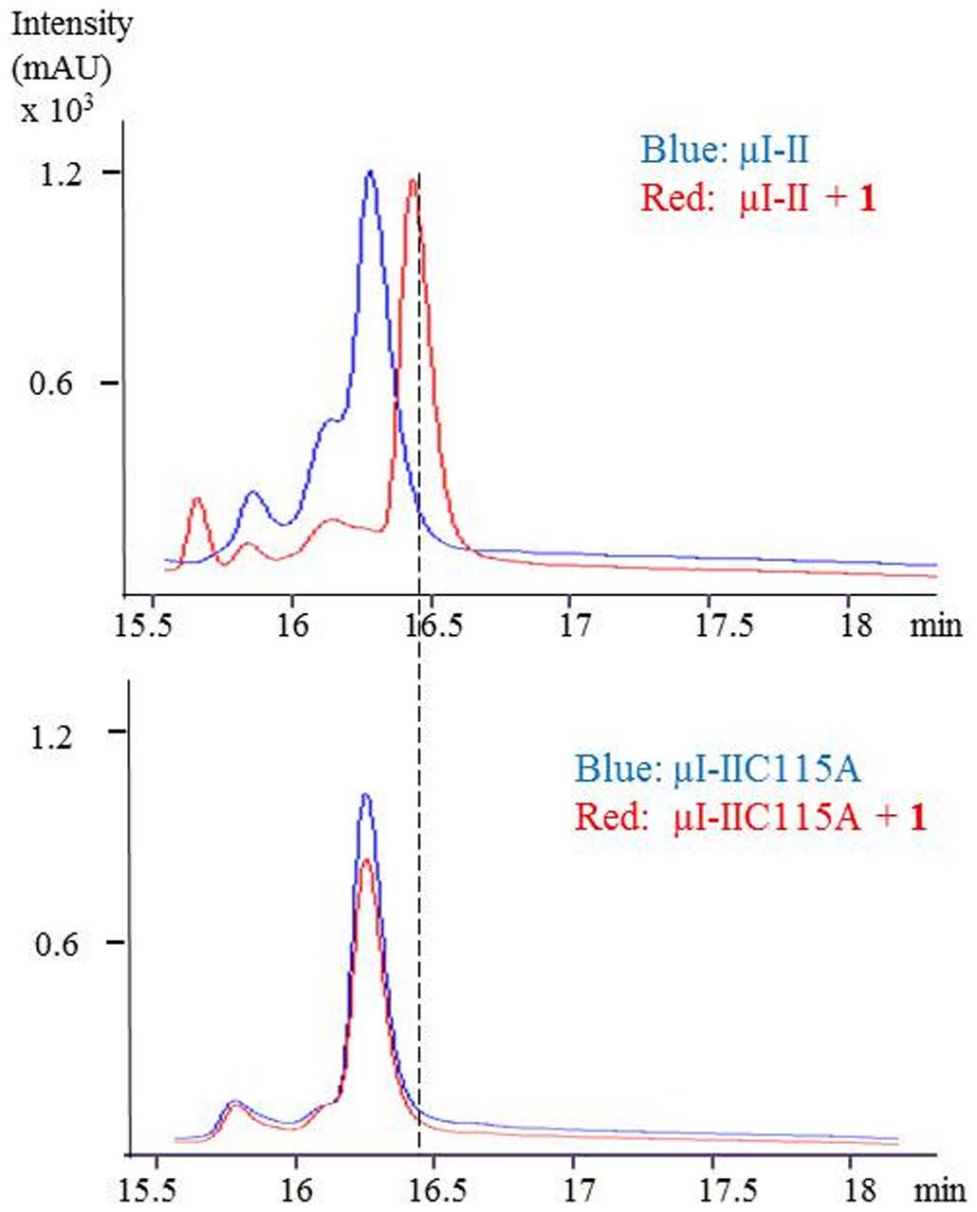
HPLC comparison of μI-II and μI-IIC115A samples after the addition of 1. The retention time of μI-II was shifted by 0.3 min, while no obvious shift can be observed in μI-IIC115A sample.

**Figure 5:**
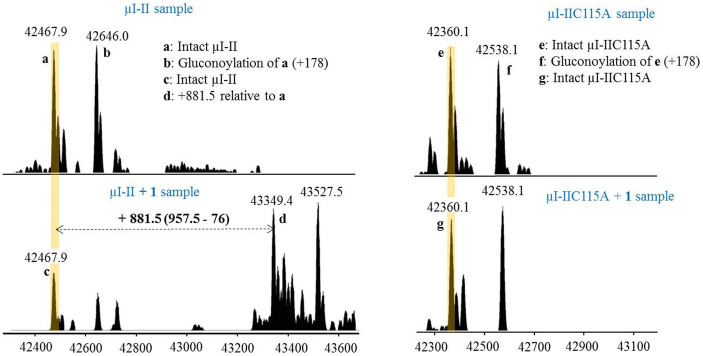
FT-ICR-MS analysis of μI-II, μI-II + 1, μI-IIC115A, and μI-IIC115A + 1. After the addition of **1**, a 881.5 shift was clearly observed in μI-II samples (peak **a**: 42467.9; peak **d**: 43349.4), while there was no shift in μI-IIC115A samples (peak **e**: 42360.1 Da; peak **g**: 42360.1 Da). Mass spectra shown are deconvoluted with neutral monoisotopic masses displayed above each peak.

**Figure 6:**
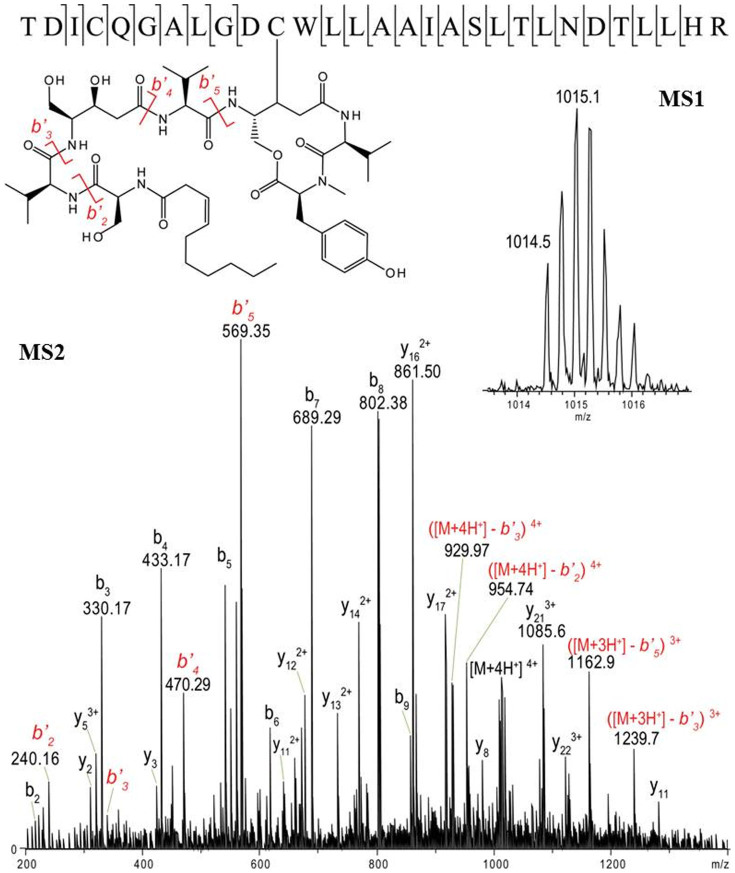
MS/MS annotation of 1-modified fragment in the μI-II + 1 sample (b ions of 1 labeled by *b*′ and red color).

We next incubated excess **1** with μI-II and μI-IIC115A, purified the samples by C4 RP-HPLC, and analyzed them by FT-ICR-MS. HPLC chromatograms showed that the retention time of μI-II was changed by 0.3 min following the addition of **1** and a shift of +881.5 amu was clearly measured by MS (see Fig. 4 and Fig. 5). This mass shift represents the displacement of β-ME (loss of 76 Da) from Cys115 and the subsequent addition of **1** (addition of 957.5 Da) in its place. On the contrary, neither a change of HPLC retention time nor a mass shift in the MS signal was found for the μI-IIC115A sample after the addition of **1** (see Fig. 4 and Fig. 5). These results clearly signify that **1** interacts specifically with the Cys115 residue. As additional confirmation of these results, both μI-II + **1** and μI-IIC115A + **1** samples were digested overnight with trypsin and analyzed by LC-MS/MS (qTOF), which allowed the detection of the **1**-modified fragment in the μI-II + **1** sample (see Fig. 6 and Supplementary Fig. 8) and not the μI-IIC115A + **1** sample. Based on these complimentary results, we conclude that thalassospiramide is exclusively associated with the Cys115 residue of calpain by a 1, 4-addition reaction.

### Molecular Modeling Study

To gain further insight into the binding properties between thalassospiramide analogues and calpain, we performed large-scale docking between various thalassospiramides and 30 representative calpain conformations (PDB file: 1ZCM) generated via molecular dynamics simulations. Our docking results showed that thalassospiramide C (**4**), which experimentally demonstrated the greatest potency, reached Cys115 over the shortest distance, had the lowest docking energy of −8.1 kcal/mol, and experienced the largest number of binding conformations with a distance of less than 5 Å (see Supplementary Table 1 and Supplementary Fig. 9). Moreover, **4** had the shortest lipopeptide side chain that enables tight interaction with the three important regions of calpain (S1, S2, and S3)^14^, which is consistent with our previous observations of markedly different bioactivities associated with the variable hydrophobic tails in the thalassospiramide series. In the case of syringolin A, which shares a similar ring structure to **4**, Clerc *et al.* found that its proteasome inhibitory activity was enhanced more than 100-fold by modifications to the side chain^29^. Furthermore, the nucleophilic attack of Cys115 leads to the *S*-configuration of the resulting chiral center, the same as the study of Cysteine Cathepsins^30^.

### Preliminary Selectivity and Toxicity Studies

Thalassospiramides **1** and **4** were inactive against the human 20S proteasome at 10 μM, and have been reported to have weak activity against papain and none against trypsin^31^,^32^. In a preliminary toxicity study, we did not observe any obvious growth inhibition against the bacterial strains *Staphylococcus aureus*, the fungal strain *Candida albicans*, and *HeLa* cells with 10 μM **1** and **4–8**. In addition, Oh *et al.* reported that at a concentration of 10 μM of either **1** or **9** or 20 μM of **10**, no cytotoxicity was observed in a mouse model^33^. These preliminary findings suggest that thalassospiramide may possess both low toxicity and good selectivity.

## Discussion

We report that thalassospiramide natural products are potent inhibitors of calpain and function differently from standard calpain inhibitors. Most inhibitors possess highly reactive aldehyde groups that bind covalently to the active site of calpain, causing disadvantages such as non-selectivity, instability, and excessive metabolism^34^,^35^,^36^,^37^. Although other examples of α,β-unsaturated amides have been reported as electrophilic inhibitors of cysteine proteases^38^,^39^,^40^, this is the first report that an α,β-unsaturated carbonyl moiety in a rigid ring system functions as an electrophilic warhead at nanomolar scale inhibitory activity against calpain. Interestingly, our previous biosynthetic study revealed that despite the great variety of side chains amongst the 16 known thalassospiramide analogues, the 12-membered macrolide ring is perfectly conserved^17^. These observations suggest that this core structure may possess important physiological function(s) and could serve as a new scaffold for drug design.

## Methods

### Fluorescence-based Calpain Activity Assay

Calpain 1 activity was measured according to the protocol provided with the Calpain Activity Assay Kit (K240-100, BioVision, USA). Tested compounds were dissolved in DMSO and serially diluted in methanol to obtain various concentrations (600 nM, 125 nM, 25 nM, 5 nM, 1 nM). The calpain inhibition assays were performed in 96-well plates. Each assay was initiated by combining 85 μL extraction buffer and 0.1 U HCAN1 (BioVision, USA). Subsequently, 1 μL test compound of the desired concentration, 10 μL reaction buffer, and 5 μL calpain substrate (Ac-LLY-AFC) was added to each well. The plate was incubated at 37°C for 1 h in the dark. The test samples were then recorded using a fluorometer equipped with an excitation wavelength of 400 nm and an emission wavelength of 505 nm. Samples without HCAN1 or test compound were defined as blank controls, and samples without test compounds were defined as negative controls. The reported IC_50_ values are the average of triplicate determinations.

### Preparation of Compounds

All thalassospiramides in this study were obtained from the marine bacteria *Thalassospira* sp. CNJ328 and *Tistrella bauzanensis* TIO7329^17^. Compounds were purified using RP HPLC (Luna 5 μ C_18 _(2), 250 × 1.00 mm, 100 Å, Phenomenex, Torrance CA) and analyzed using UPLC-ESI-HRMS (1.7 μm C_18_ column, 0.25 mL/min, gradient from 5% to 95% CH_3_CN with 0.1% TFA, 25 min). The NMR spectra were obtained using a Varian Inova 500 MHz spectrometer. All samples were dissolved in MeOH-*d*_4_, and chemical shifts were reported in ppm relative to TMS. The ESI-HRMS analysis was conducted using a Bruker Daltonics micrOTOF instrument (Bruker Daltonics GmbH, Bremen, German). The procedures of chemical modifications of **1** were described: (a) Ester hydrolysis of 12-membered ring of **1**. Compound **1** (1 mg) was dissolved in 3 mL water solution (0.01 N NaOH, 50% methanol) and stirred for 2 hour at room temperature. After the reaction, the sample was neutralized and the solution was evaporated *in vacuo*. The product **2** (0.6 mg) was dissolved in MeOH and purified using RP-HPLC with an isocratic method of 45% CH_3_CN in water. (b) Hydrogenation of double bond of **1.** Compound **1** was dissolved in ethanol and stirred for 5 hour together with 10% Pd/C catalyst under atmospheric H_2_ at room temperature. The catalyst was blocked with 0.25-μm filter membrane and the solvent was removed *in vacuo*. The reaction product was confirmed by an increased molecular weight of 4 daltons and MS/MS annotation by LC/MS analysis.

### MALDI-TOF Mass Spectrometry Analysis

For sample preparation, the C4 Zip Tip (Millipore) was used for desalting and purification by washing with 20% acetonitrile water solution (0.1% TFA). Protein samples were eluted with 60% acetonitrile water solution (0.1% TFA) and dried under a stream of nitrogen. Treated proteins were dissolved in a saturated sinapinic acid (SA) matrix solution and applied to the MALDI ground steel plate (Bruker Daltonics). After air drying, the samples were analyzed in linear mode using an ultrafleXtreme MALDI-TOF-MS (Bruker Daltonics, Germany).

### Trypsin Digestion for Free HCAN1, HCAN1 + 1 and HCAN1 + 3 Samples

Trypsin (mass spectrometry grade, Promega) was added to the 3 samples for digestion to a final proteinase: protein ratio of 1:50 (w/w) and incubated at 37°C for 24 hours. Digested samples were then desalted and purified using the C18 Zip Tip (Millipore), dissolved in saturated SA matrix solution, and analyzed in reflectron mode and linear mode by MALDI-TOF/TOF-MS.

### Expression, Purification and Identification of μI-II and μI-IIC115A

The cDNAs were obtained from *HeLa* cells by reverse transcription (SuperScript® III Reverse Transcriptase, Invitrogen™), and human μI-II was amplified by PCR using *pfu* DNA polymerase (Tiangen, China). The active site Cys115 was mutated to Ala by overlap PCR and confirmed by sequencing. The two DNA sequences were inserted into plasmid pet28a using *BamHI* and *SalI*, and the recombinant plasmids were sequenced and BLAST against human calpain 1 (see Supplementary Fig. 2).

μI-II and μI-IIC115A were expressed in *E. coli* BL21, and grown on LB medium containing 30 μg/mL kanamycin at 37°C with shaking (225 rpms). Protein expression was induced by the addition of 0.5 mM IPTG at a bacterial concentration of OD_600_ 0.6, followed by bacterial cell growth for an additional 18 h at 16°C before harvesting. Protein purification was conducted using Ni^2+^-affinity columns according to the manufacturer's instructions. Eluted protein was concentrated using a 30 K molecular weight filter and exchanged into storage buffer (20 mM imidazole-HCl, 5 mM β-mercaptoethanol, 1 mM EDTA, 1 mM EGTA, 30% glycol, pH 7.6).

The two expressed proteins were identified by SDS-PAGE and MS/MS annotation of trypsin digests. The map of SDS-PAGE showed that the molecular weight of two expressed proteins were 35–48 kDa (see Supplementary Fig. 3), consistent with the predicted molecular weight. Tryptic peptides of μI-II and μI-IIC115A were analyzed by LC-HR-MS/MS using a Bruker Daltonics micrOTOF-QII. The tryptic peptide coverage of each protein, as determined by MS^1^ and verified by MS^2^, and the annotation of active site containing peptides are shown in Supplementary Fig. 4 and Fig. 5.

### Intact Protein Sample Preparation and Analysis by FT-ICR-MS

In preparation for FT-ICR-MS analysis, the 4 proteins samples (μI-II, μI-II + **1**, μI-IIC115A, and μI-IIC115A + **1**) were purified by off-line HPLC (Agilent Infinity 1200 equipped with a multiple wavelength detector). Stock protein samples were thawed and then mixed with a 1 mM CaCl_2 _solution. A portion of μI-II and a portion of μI-IIC115A were each mixed with a molar excess of **1** and these were allowed to incubate at room temperature for an additional 10 minutes prior to HPLC purification. Samples, each containing 10 μg of protein, were injected onto a C_4_ RP-HPLC column (Jupiter 5 μ C_4_, 150 mm × 4.60 mm, 300 Å, Phenomenex). These samples were loaded at 1 ml/min of 90% mobile phase A (H_2_O + 0.1% TFA) and 10% mobile phase B (ACN + 0.1% TFA), which was held for 5 minutes, then eluted by increasing to 90% mobile phase B over 20 minutes. Absorbance at 220 nm was monitored and fractions of the HPLC eluent containing the major peaks were collected, flash frozen, and lyophilized to dryness. Immediately prior to FT-ICR-MS analysis, lyophilized fractions were dissolved in 100 μl of a solution containing a mixture of 49.5% H_2_O, 49.5% methanol, and 1% formic acid (all LC/MS grade). Samples were introduced into an LTQ-FT hybrid mass spectrometer with a 6.4 T magnet (Thermo Electron, North America) using a TriVersa NanoMate (Advion BioSciences, Inc.). The NanoMate was utilized in direct-infusion mode with a spray pressure of 0.3 psig and an ESI voltage of 1.4 kV. The LTQ-FT ion optics were first tuned to m/z 816 using cytochrome C. The LTQ capillary temperature was maintained at 200°C. For the acquisition of high-resolution FT-ICR-MS spectra of the intact protein samples the following settings were used: 200000 resolution, m/z range of 700–1400, 3 micro-scans per scan, max injection time of 8000 ms, and an FT automatic gain control (AGC) of 8 e5. In order to improve the clarity of the FT-ICR measurements of the intact proteins, an isolation window of 100 m/z units was centered upon m/z 1015 in order to capture multiple charge states of the protein within each of the four samples, and a minimum of 1000 scans were acquired and averaged (see Supplementary Fig. 7). Deconvolution of intact protein FT-ICR mass spectra was performed by using Xtract (Thermo Electron, Bremen, Germany).

### Limited Trypsin Digestion and FT-ICR-MS Analysis of μI-II

A sample of protein μI-II was briefly digested using trypsin (Trypsin Singles, proteomics grade, Sigma-Aldrich). A final trypsin:protein ratio of 1:20 (w/w) was mixed and incubated at 37°C for 30 minutes in 40 mM ammonium bicarbonate buffer. The reducing agents DTT and TCEP were intentionally excluding as these reducing agents have been shown to remove β-ME from the cysteines^27^. The limited trypsin digest of μI-II was fractionated by HPLC using the same column and gradient that was utilized for the intact protein analysis. The eluent was collected in 1 ml fractions (1 min/fraction), flash frozen, and lyophilized. The collected fractions were dissolved in 30–60 μL of the 49.5% H_2_O, 49.5% methanol, and 1% formic acid mixture and analyzed by FT-MS using similar instrument parameters as previously outlined, with the exceptions being that the FT-ICR resolution was lowered to 50000 and the range of m/z 200–2000 was recorded for all fractions. Peptides were identified on the basis of their intact mass and β-ME modified peptides were verified by MS/MS fragmentation (see Supplementary Fig. 6).

### Bottom-up Analysis of Protein Samples by LC-HR-MS/MS

In preparation for bottom-up analysis of the 4 proteins samples (μI-II, μI-II + **1**, μI-IIC115A, and μI-IIC115A + **1**) each one was reduced with DTT, alkylated, and incubated with a trypsin: protein ration of 1:20 (w/w) at 37°C for 18 hours. Samples were subsequently quenched with an equal volume of 10% formic acid. For LC-HR-MS/MS analysis and Agilent Infinity 1290 UPLC connected to a Bruker Daltonics micrOTOF-QII qTOF mass spectrometer. The trypsin digested samples were separated using an RP-C_18_ column (Luna 3 μ C_18 _(2), 100 mm × 2.0 mm, 100 Å, Phenomenex). Samples were loaded at 0.25 ml/min of 95% mobile phase A (100% H_2_O + 0.1% formic acid) and 5% mobile phase B (ACN + 0.1% formic acid), which was held for 3 minutes, then mobile phase B was increased to 65% over 30 minutes, and then B was increased to 100% over 2 minutes. The micrOTOF-QII was configured to have a nebulizer pressure of 1.6 bar, dry gas temperature of 200°C, dry gas flow-rate of 7 L/min. Mass spectra were acquired with an MS^1^ spectral rate of 2 Hz, an MS^2^ rate of 3 Hz, and the collision RF was stepped from 200 Vpp to 600 Vpp (increments of 100 V) during each MS^2^ event in order to maximize observed fragment ions. A "lock-mass" internal calibrant (m/z 922.01; CAS 58943-98-9) was continuously introduced into the mass spectrometer in order to maintain proper calibration throughout the run. The MS/MS annotation of thaA-modified peptide is detailed in Supplementary Fig. 8.

### Molecular Dynamics Simulations and Docking Study of Calpain and Thalassospiramides

The currently available structures for calpain-1 are 1ZCM and 2ARY, from which we selected the former because it is a bound structure in which the ligand is covalently linked to Cys115^28^. To prepare the MD simulations, we removed the ligand and determined the protonation states of all histidines using the PDB2PQR server^41^. The protein was then solvated in a water box with 13309 SPC waters^42^ and 9 sodium ions to neutralize the system. The GROMACS 4.5.5 simulation package^43^ with the Amber99sb force field^44^ were used in the MD simulations due to their speed. The system was minimized with a steepest descent algorithm followed by 200 ps MD simulations with a position restraint for heavy protein atoms, under NPT conditions with 1 bar of pressure and a temperature of 310 K using a V-rescale thermostat^45^. The cut-offs for both VDW and short-range electrostatic interactions were set at 10 Å, and long-range electrostatic interactions were treated according to the Particle-Mesh Ewald method^46^. Water molecules were constrained using the SETTLE algorithm^47^, and all protein bonds were constrained by the LINCS algorithm^48^. Finally, we performed three independent 50-ns NPT simulations with the same initial conformation (i.e., the last frame of the position restraint simulation) and different initial velocities. The conformations were saved every 5 ps.

All of the 30,000 MD simulation frames were collected and divided into 30 clusters according to the C-alpha of two flexible loops (residue 69–82 and 251–261) using the k-center algorithm^49^. The average RMSD within each cluster was approximately 1.5 Å. We chose one representative conformation from each cluster for flexible docking using AutoDock Vina.

We used AutoDock Tools (ADT) to prepare the proteins and ligands prior to docking. The ADT merged nonpolar hydrogens of proteins and automatically detected each bond type of the ligands. Proteins and ligands were added with Gasteiger partial charges^50^. AutoDock Vina, which significantly improved the average accuracy of the binding mode prediction compared to AutoDock 4, was applied for the docking study. The docking grid box was centered near Cys115 with a volume of 24*26*34 (A^3^), and each docking had 48 (parameter: exhaustive) parallel runs. The sequence index of the selected flexible residues was: 72, 79, 109, 115, 254, 260, 261, 272 and 298^51^.

### Fluorescence-based 20S Proteasome Inhibition Assay

Thalassospiramide **1** and **4** were serially diluted in methanol to obtain various concentrations, and then incubated with 1 nM 20S proteasome in 96-well plates (Tris 25 mM, pH 7.5; SDS 0.03%; EDTA 0.5 mM, final reaction volume 40 μL) for 15 min at 37°C. The specific fluorogenic proteasome substrate Suc-LLVY-AMC (10 μL, 200 mM) was added and incubated for another 15 min at 37°C, and then the fluorescence in each well was measured. The assay wells without inhibitor were conducted as positive control (related to 100% of enzyme activity)^52^.

### Preliminary Toxicity Assays

The preliminary toxicity assays included antimicrobial and cytotoxic assays. In antimicrobial assay, LB (10 g of tryptone, 5 g of yeast extract, 10 g of NaCl, 1 L of dd H_2_O) and potato dextrose medium (200 g of potato, 20 g of dextrose, 1 L of dd H_2_O) were used to inoculate strain *S. aureus* ATCC 43300 and *C. albicans* ATCC 76615 (28°C, for 12 h), respectively. The test samples were prepared at 1 mM in DMSO and added to the broth (96-well plate) at 1% (V/V, final concentration 10 μM). Then, the microbes were incubated at 28°C overnight. Cell growth was recorded by measuring the optical density at 600 nm, and Penicillin (for *S. aureus*) and Natamycin (for *C. albicans*) were used as positive controls. Human *HeLa* cells were used in cytotoxic assay. Cells (80 μL, 1 × 10^5^/mL) were planted into 96-well plates for 12 h, then the test samples were dissolved in DMSO and added to the assay medium to inoculate for another 48 h. The cell viability was assayed by the MTT method.

## Author Contributions

L.L., M.M., Z.C., X.H., P.D., Y.X., B.M. and P.Q. designed the experiments, L.L, M.M., S.G., W.Z., G.Z., L.L.(IU). executed the experiments, L.L., M.M., S.G. analyzed data and prepared the figures, and L.L. wrote the manuscript with the input from all authors.

## Supplementary Material

Supplementary Information supporting information

## Figures and Tables

**Figure 1:**
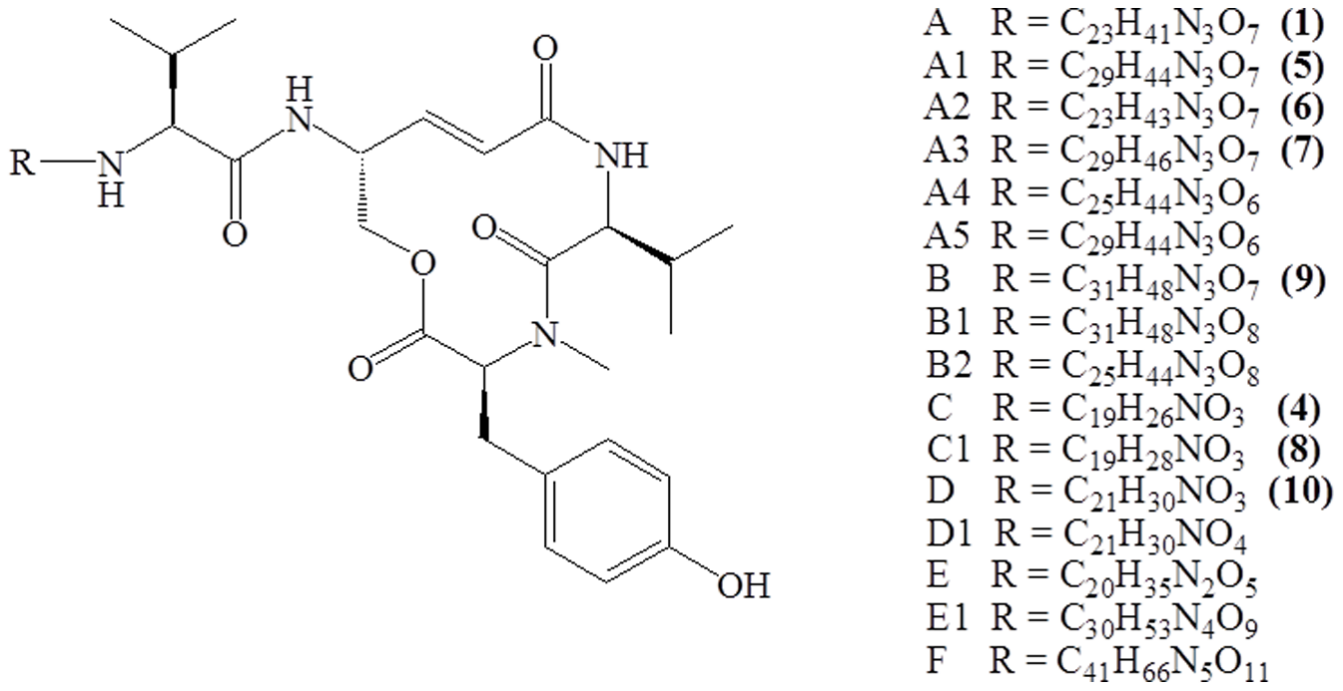
Chemical structure of thalassospiramide analogues. All thalassospiramides share a rigid 12-membered ring and a variable lipopeptide side chain (R). See Ross et al.^17^ for full structures.

**Table 1:** Inhibitory activity of thalassospiramides against HCAN1

Compound	IC_50_ (nM)
Thalassospiramide A (**1**)	56.5 ± 2.4
Thalassospiramide A1 (**5**)	42.3 ± 2.3
Thalassospiramide A2 (**6**)	58.2 ± 3.1
Thalassospiramide A3 (**7**)	47.5 ± 2.9
Thalassospiramide A4	41.3 ± 3.4
Thalassospiramide A5	22.2 ± 3.0
Thalassospiramide B (**9**)	29.0 ± 2.0
Thalassospiramide B1	78.8 ± 4.1
Thalassospiramide B2	69.4 ± 4.3
Thalassospiramide C (**4**)	3.4 ± 1.2
Thalassospiramide C1 (**8**)	4.8 ± 1.8
Thalassospiramide D (**10**)	21.2 ± 3.7
Thalassospiramide D1	34.7 ± 2.5
Thalassospiramide E	35.0 ±.2.2
Thalassospiramide E1	20.9 ± 1.6
Thalassospiramide F	12.5 ± 3.4
