# Supplementary material for: Mechanism of Action of Thalassospiramides, A New Class of Calpain Inhibitors
Source: Sci Rep. 2015 Mar 5;5:8783. doi: 10.1038/srep08783 (PMC4350077; doi:10.1038/srep08783)
Supplement: Supplementary Information — supporting information [file srep08783-s1.pdf]

Supporting information

Mechanism of Action of Thalassospiramides, A New Class of Calpain Inhibitors

Liang Lu,[1] Michael J. Meehan,[2] Shuo Gu,[3] Zhilong Chen,[3] Weipeng Zhang,[1] Gen Zhang,[1] Lingli Liu,[1] Xuhui Huang,[3] Pieter C. Dorrestein,[2, 4, 5] Ying Xu,[*, 1, 6] Bradley S. Moore, [*, 2, 5] Pei-Yuan Qian[*, 1]

[1] KAUST Global Collaborative Research, Division of Life Science, School of Science, Hong Kong University of Science and Technology, Clear Water Bay, Hong Kong, China

[2] Skaggs School of Pharmacy & Pharmaceutical Sciences, University of California at San Diego, La Jolla, California 92037, United States

[3] Department of Chemistry, Hong Kong University of Science and Technology, Clear Water Bay, Hong Kong, China

[4] Department of Pharmacology, University of California at San Diego, La Jolla, California 92037, United States

[5] Center for Marine Biotechnology and Biomedicine, Scripps Institution of Oceanography, University of California at San Diego, La Jolla, California 92037, United States

[6] School of Life Science, Shenzhen University, Nanhai Ave 3688, Shenzhen, Guangdong Province, 518060, China

* Correspondence should be addressed to P.Y.Qian (boqianpy@ust.hk), B. Moore (bsmoore@ucsd.edu) or Y. Xu (boxuying@szu.edu.cn).

Table of contents

Supplementary Figures

Supplementary Fig. 1: MS/MS analysis of peak 3097.5 (peptide 105-133) by MALDI-TOF/TOF.

Supplementary Fig. 2: Sequence alignment of human calpain 1 and two expressed proteins in *E.coli*.

Supplementary Fig. 3: SDS-PAGE of μI-II and μI-IIC115A.

Supplementary Fig. 4: Identification of μI-II by MS/MS annotation with LC-HR-MS/MS (qTOF).

Supplementary Fig. 5: Identification of μI-IIC115A by MS/MS annotation with LC-HR-MS/MS (qTOF).

Supplementary Fig. 6: MS/MS annotation of β-ME modified peptides in μI-II by FT-ICR-MS.

Supplementary Fig. 7: FT-ICR-MS data comparison of μI-II and μI-IIC115A samples.

Supplementary Fig. 8: MS/MS annotation of **1**-modified peptide by HR-MS/MS (qTOF).

Supplementary Fig. 9: Representative pose of **4** (thalassospiramide C) in the calpain model.

Supplementary Fig. 10: Characterization of **1** by HRMS and ^{1}H NMR.

Supplementary Fig. 11: Characterization of **2** by HRMS and MS^n.

Supplementary Fig. 12: Characterization of **3** by HRMS and MS^n.

Supplementary Fig. 13: Characterization of **4** by HRMS and H-NMR.

Supplementary Table

Supplementary Table 1: Docking results for the representative thalassospiramides.

Supplementary Figures

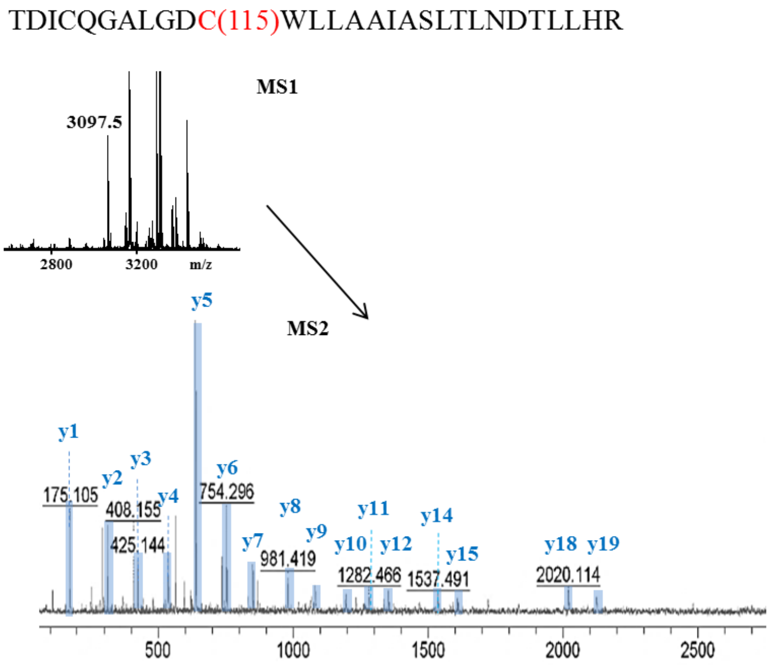

Species	Cal. mass	Obs. mass	Error (Da)
y1	175.12	175.11	0.01
y2	312.18	312.11	0.07
y3	425.26	425.14	0.12
y4	538.35	538.21	0.14
y5	639.39	639.27	0.12
y6	754.42	754.30	0.12
y7	868.46	868.35	0.11
y8	981.55	981.42	0.13
y9	1082.6	1082.5	0.1
y10	1195.7	1195.6	0.1
y11	1282.7	1282.5	0.2
y12	1353.8	1353.6	0.2
y14	1537.9	1537.5	0.4
y15	1608.9	1608.6	0.3
y18	2021.2	2021.1	0.1
y19	2124.2	2124.0	0.2

Supplementary Fig. 1 | MS/MS analysis of peak 3097.5 (peptide105-133) by MALDI-TOF/TOF (LIFT mode).

```
µI-II            MGSSHHHHHHSSGLVPRGSHMASMTGGQQMGRGSGRHENAIKYLGQDYEQLRVRCLQSGT
µI-IIC115A       MGSSHHHHHHSSGLVPRGSHMASMTGGQQMGRGSGRHENAIKYLGQDYEQLRVRCLQSGT
Human calpain 1                                    GRHENAIKYLGQDYEQLRVRCLQSGT

µI-II            LFRDEAFPPVPQSLGYKDLGPNSSKTYGIKWKRPTELLSNPQFIVDGATRTDICQGALGD
µI-IIC115A       LFRDEAFPPVPQSLGYKDLGPNSSKTYGIKWKRPTELLSNPQFIVDGATRTDICQGALGD
Human calpain 1  LFRDEAFPPVPQSLGYKDLGPNSSKTYGIKWKRPTELLSNPQFIVDGATRTDICQGALGD

µI-II            CWLLAAIASLTLNDTLLHRVVPHGQSFQNGYAGIFHFQLWQFGEWVDVVVDDLLPIKDGK
µI-IIC115A       AWLLAAIASLTLNDTLLHRVVPHGQSFQNGYAGIFHFQLWQFGEWVDVVVDDLLPIKDGK
Human calpain 1  CWLLAAIASLTLNDTLLHRVVPHGQSFQNGYAGIFHFQLWQFGEWVDVVVDDLLPIKDGK

µI-II            LVFVHSAEGNEFWSALLEKAYAKVNGSYEALSGGSTSEGFEDFTGGVTEWYELRKAPSDL
µI-IIC115A       LVFVHSAEGNEFWSALLEKAYAKVNGSYEALSGGSTSEGFEDFTGGVTEWYELRKAPSDL
Human calpain 1  LVFVHSAEGNEFWSALLEKAYAKVNGSYEALSGGSTSEGFEDFTGGVTEWYELRKAPSDL

µI-II            YQIILKALERGSLLGCSIDISSVLDMEAITFKKLVKGHAYSVTGAKQVNYRGQVVSLIRM
µI-IIC115A       YQIILKALERGSLLGCSIDISSVLDMEAITFKKLVKGHAYSVTGAKQVNYRGQVVSLIRM
Human calpain 1  YQIILKALERGSLLGCSIDISSVLDMEAITFKKLVKGHAYSVTGAKQVNYRGQVVSLIRM

µI-II            RNPWGEVEWTGAWSDSSSEWNNVDPYERDQLRVKMEDGEFWMSFRDFMREFTRLEICNLT
µI-IIC115A       RNPWGEVEWTGAWSDSSSEWNNVDPYERDQLRVKMEDGEFWMSFRDFMREFTRLEICNLT
Human calpain 1  RNPWGEVEWTGAWSDSSSEWNNVDPYERDQLRVKMEDGEFWMSFRDFMREFTRLEICNLT

µI-II            PDLDKLAAALEHHHHHH
µI-IIC115A       PDLDKLAAALEHHHHHH
Human calpain 1  PD
```

Supplementary Fig. 2 | Sequence alignment of human calpain 1 and two expressed proteins. The sequence of human calpain 1 was downloaded from *pubmed* (accession: 2ARY_A). For µI-IIC115A protein, the active site Cys115 was mutated to Ala (marked in red).

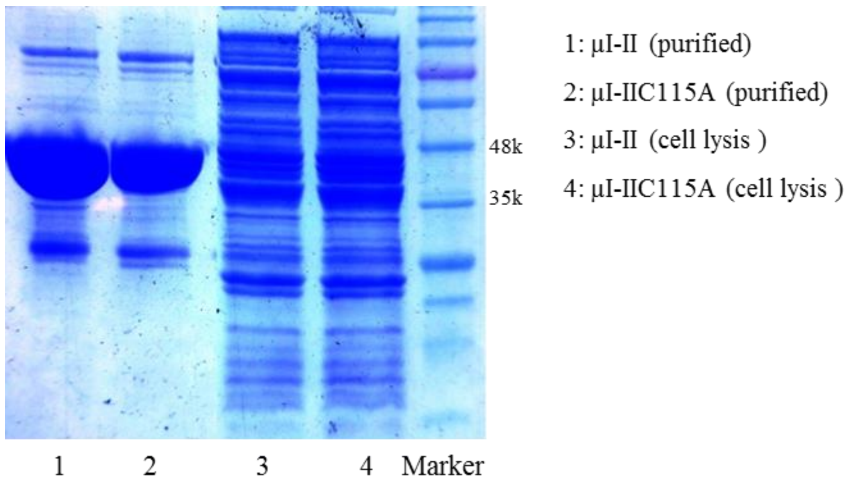

Supplementary Fig. 3 | SDS-PAGE of μI-II and μI-IIC115A protein (purified/ in the cell lysis).

A.

AA sequence coverage of μI-II:

GSSHHHHHHSSGLVPRGSHMASMTGGQQMGRGSGRHENAIKYLGQDYEQLRVRCLQSGTLFRDEAFPPVPQSLGY
KDLGPNSSKTYGIKWKRPTELLSNPQFIVDGATRTDICQGALGDC(115)WLLAAIASLTLNDTLLHRVVPHGQSFQNG
YAGIFHFQLWQFGEWVDVVVDDLLPIKDGKLVFVHSAEGNEFWSALLEKAYAKVNGSYEALSGGSTSEGFEDFTG
GVTEWYELRKAPSDLYQIILKALERGSLLGCSIDISSVLDMEAITFKKLVKGHAYSVTGAKQVNYRGQVVSLIRMRN
PWGEVEWTGAWSDSSSEWNNVDPYERDQLRVKMEDGEFWMSFRDFMREFTRLEICNLTPDLDKLAAALEHHHHH
H

Start	End	Amino acid sequence	Charge	Cal. Mass	Obs. Mass	Error(Da)
17	31	(R) GSHMASMTGGQQMGR(G)	3	512.56	512.56	0.00
17	31	(R) GSHMASMTGGQQMGR(G)	2	768.33	768.33	0.00
42	51	(K) YLGQDYEQLR(V)	2	642.82	642.82	0.00
54	62	(R) CLQSGTLFR(D)	2	512.77	512.77	0.00
63	76	(R) DEAFPPVPQSLGYK(D)	3	516.60	516.60	0.00
63	76	(R) DEAFPPVPQSLGYK(D)	2	774.39	774.40	-0.01
85	89	(K) TYGIK(W)	2	291.17	291.17	0.00
85	89	(K) TYGIK(W)	1	581.33	581.33	0.00
92	109	(K) RPTELLSNPQFIVDGATR(T)	3	672.03	672.03	0.00
110	138	(R) TDICQGALGDCWLLAAIASLTLNDTLLHR(V)	3	1033.2	1033.2	0.0
139	176	(R) VVPHGQSFQNGYAGIFHFQLWQFGEWVDVVVDDLLPIK(D)	4	1097.1	1097.1	0.0
180	198	(K) LVFVHSAEGNEFWSALLEK(A)	3	726.04	726.05	-0.01
203	233	(K) VNGSYEALSGGSTSEGFEDFTGGVTEWYELR(K)	4	836.88	836.88	0.00
203	233	(K) VNGSYEALSGGSTSEGFEDFTGGVTEWYELR(K)	3	1115.5	1115.5	0.0
235	245	(K) APSDLYQIILK(A)	2	630.86	630.87	-0.01
250	271	(R) GSLLGCSIDISSVLDMEAITFK(K)	3	767.06	767.06	0.00
291	298	(R) GQVVSLIR(M)	2	436.27	436.28	-0.01
301	327	(R) NPWGEVEWTGAWSDSSSEWNNVDPYER(D)	4	800.09	800.10	-0.01
301	327	(R) NPWGEVEWTGAWSDSSSEWNNVDPYER(D)	3	1066.5	1066.5	0.0
334	344	(K) MEDGEFWMSFR(D)	2	717.80	717.80	0.00
353	364	(R) LEICNLTPDLDK(L)	2	687.35	687.36	-0.01

Supplementary Fig. 4 | Identification of μI-II by MS/MS annotation with LC-HR-MS/MS (qTOF). A) Amino acid sequence coverage of μI-II and m/z's of all tryptic peptides verified by MS^2.

B.

T D I C Q G A L G D C W L L A A I A S L T L N D T L L H R

MS1

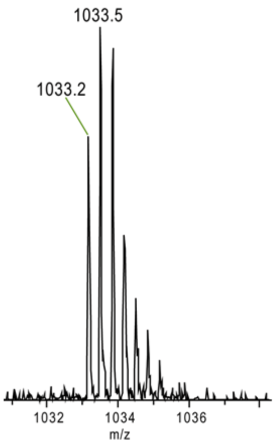

Species	Cal. mass	Obs. mass	Error(Da)	Species	Cal. mass	Obs. mass	Error(Da)
b2	217.08	217.08	0.00	y18^{+2}	1011.1	1011.1	0.0
b3	330.17	330.17	0.00	y18^{+2}	1062.6	1062.6	0.0
b4	433.18	433.18	0.00	y20^{+2}	1120.1	1120.1	0.0
b5	561.23	561.24	-0.01	y21^{+2}	1148.6	1148.6	0.0
b6	618.26	618.25	0.01	y22^{+2}	1205.2	1205.2	0.0
b7	689.29	689.29	0.00	y23^{+2}	1240.7	1240.7	0.0
b8	802.38	802.38	0.00	y24^{+2}	1269.2	1269.2	0.0
y2	312.18	312.18	0.00	y25^{+2}	1333.2	1333.2	0.0
y3	425.26	425.26	0.00	y25^{+2}	889.14	889.13	0.01
y4	538.35	538.36	-0.01	y26^{+2}	1384.7	1384.7	0.0
y12^{+2}	677.38	677.38	0.00	y26^{+2}	923.48	923.47	0.01
y16^{+2}	861.50	861.50	0.00	y27^{+2}	961.17	961.18	-0.01

MS2

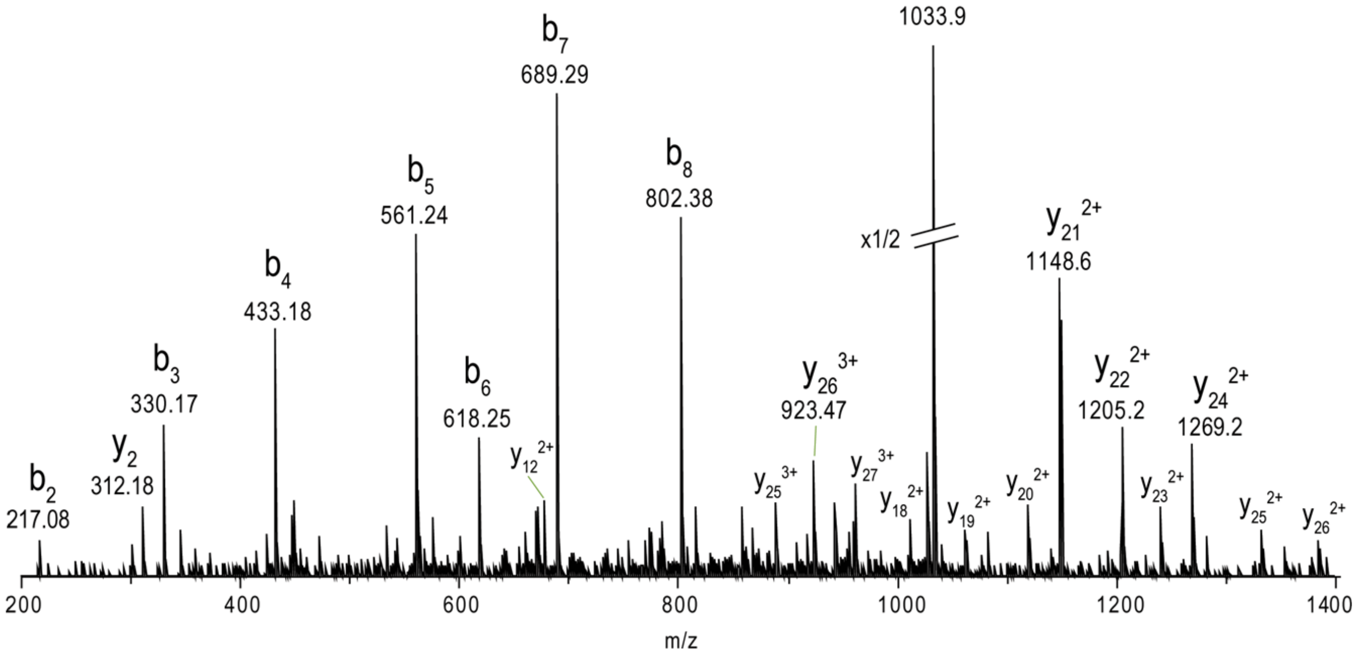

Supplementary Fig. 4 | Identification of μI-II by MS/MS annotation with LC-HR-MS/MS (qTOF). B) MS/MS annotation of peptide TDICQGALGDCWLLAAIASLTLNDTLLHR (m/z=1033.2, +3 charge).

A.

AA sequence coverage of μI-IIC115A :

GSSHHHHHHSSGLVPRGSHMASMTGGQQMGRGSGRHENAIKYLGQDYEQLRVRCLQSGTLFRDEAFPPVPQSLGYKDLGP
NSSKTYGIKWKRPTELLSNPQFIVDGATRTDICQGALGDA(115)WLLAAIASLTLNDTLLHRVVPHGQSFQNGYAGIFHFQLW
QFGEWVDVVVDDLLPIKDGKLVFVHSAEGNEFWSALLEKAYAKVNGSYEALSGGSTSEGFEDFTGGVTEWYELRKAPSDL
YQIILKALERGSLLGCSIDISSVLDMEAITFKKLVKGHAYSVTGAKQVNYRGQVVSLIRMRNPWGEVEWTGAWSDSSSEWN
NVDPYERDQLRVKMEDGEFWMSFRDFMREFTRLEICNLTPDLDKLAAALEHHHHHH

Start	End	Amino acid sequence	Charge	Cal. Mass	Obs. Mass	Error(Da)
17	31	(R) GSHMASMTGGQQMGR(G)	3	512.56	512.56	0.00
17	31	(R) GSHMASMTGGQQMGR(G)	2	768.33	768.34	-0.01
42	51	(K) YLGQDYEQLR(V)	2	642.82	642.82	0.00
54	62	(R) CLQSGTLFR(D)	2	512.77	512.76	0.01
63	76	(R) DEAFPPVPQSLGYK(D)	3	516.60	516.60	0.00
63	76	(R) DEAFPPVPQSLGYK(D)	2	774.39	774.39	0.00
77	84	(K) DLGPNSSK(T)	2	409.21	409.21	0.00
85	89	(K) TYGIK(W)	2	291.17	291.17	0.00
85	89	(K) TYGIK(W)	1	581.33	581.33	0.00
92	109	(K) RPTELLSNPQFIVDGATR(T)	3	672.03	672.03	0.00
92	109	(K) RPTELLSNPQFIVDGATR(T)	2	1007.5	1007.5	0.0
110	138	(R) TDICQGALGDAWLLAAIASLTLNDTLLHR(V)	3	1022.5	1022.5	0.0
139	176	(R) VVPHGQSFQNGYAGIFHFQLWQFGEWVDVVVDDLLPIK(D)	4	1097.1	1097.1	0.0
180	198	(K) LVFVHSAEGNEFWSALLEK(A)	3	726.04	726.04	0.00
203	233	(K) VNGSYEALSGGSTSEGFEDFTGGVTEWYELR(K)	4	836.88	836.93	-0.05
203	233	(K) VNGSYEALSGGSTSEGFEDFTGGVTEWYELR(K)	3	1115.5	1115.5	0.0
235	245	(K) APSDLYQIILK(A)	2	630.86	630.87	-0.01
250	271	(R) GSLLGCSIDISSVLDMEAITFK(K)	3	767.06	767.06	0.00
291	298	(R) GQVVSLIR(M)	2	436.27	436.28	0.00
301	327	(R) NPWGEVEWTGAWSDSSSEWNNVDPYER(D)	4	800.09	800.10	-0.01
301	327	(R) NPWGEVEWTGAWSDSSSEWNNVDPYER(D)	3	1066.5	1066.5	0.0
334	344	(K) MEDGEFWMSFR(D)	2	717.80	717.80	0.00
345	348	(R) DFMR(E)	1	568.26	568.25	0.01
353	364	(R) LEICNLTPDLDK(L)	2	687.35	687.36	-0.01

Supplementary Fig. 5 | Identification of μI-IIC115A by MS/MS annotation with LC-HR-MS/MS (qTOF). A) Amino acid sequence coverage of μI-IIC115A and m/z's of all tryptic peptides verified by MS^2.

B.

T D I C Q G A L G D A W L L A A I A S L T L N D T L L H R

MS1

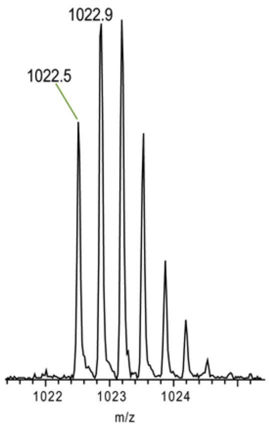

Species	Cal. mass	Obs. mass	Error(Da)	Species	Cal. mass	Obs. mass	Error(Da)
b2	217.08	217.08	0.00	y9^{+2}	541.80	541.80	0.00
b3	330.17	330.17	0.00	y11^{+2}	641.86	641.86	0.00
b4	433.18	433.18	0.00	y12^{+2}	677.38	677.38	0.00
b5	561.23	561.23	0.00	y13^{+2}	733.92	733.92	0.00
b6	618.26	618.26	0.00	y10	1195.7	1195.7	0.0
b7	689.29	689.29	0.00	y11	1282.7	1282.7	0.0
b7^{+2}	345.15	345.16	-0.01	y12	1353.8	1353.7	0.1
b8	802.38	802.38	0.00	y14	1537.9	1537.9	0.0
b9	859.40	859.40	0.00	y14^{+2}	769.44	769.43	0.01
b10	974.43	974.42	0.01	y15^{+2}	804.96	804.96	0.00
b11	1045.5	1045.5	0.0	y16^{+2}	861.50	861.50	0.00
b13^{+2}	672.82	672.82	0.00	y17^{+2}	918.04	918.04	0.00
b14^{+2}	729.36	729.36	0.00	y18^{+2}	1011.1	1011.1	0.0
b15^{+2}	764.88	764.88	0.00	y19^{+2}	1046.6	1046.6	0.0
b16^{+2}	800.40	800.40	0.00	y20^{+2}	1104.1	1104.1	0.0
b17^{+2}	856.94	856.93	0.01	y21^{+2}	1132.6	1132.6	0.0
b18^{+2}	892.46	892.45	0.01	y21^{+2}	755.42	755.42	0.00
y2	312.18	312.18	0.00	y22^{+2}	1189.2	1189.2	0.0
y3	425.26	425.26	0.00	y23^{+2}	1224.7	1224.7	0.0
y4	538.35	538.35	0.00	y24^{+2}	1253.2	1253.2	0.0
y5	639.39	639.40	-0.01	y25^{+2}	1317.2	1317.2	0.0
y6	754.42	754.42	0.00	y25^{+3}	878.49	878.49	0.00
y7	868.46	868.46	0.01	y26^{+2}	1368.7	1368.7	0.0
y8	981.55	981.54	0.01	y26^{+3}	912.82	912.83	-0.01
y9	1082.6	1082.6	0.0	y27^{+3}	950.52	950.53	-0.01
				y28^{+3}	988.86	988.87	-0.01

MS2

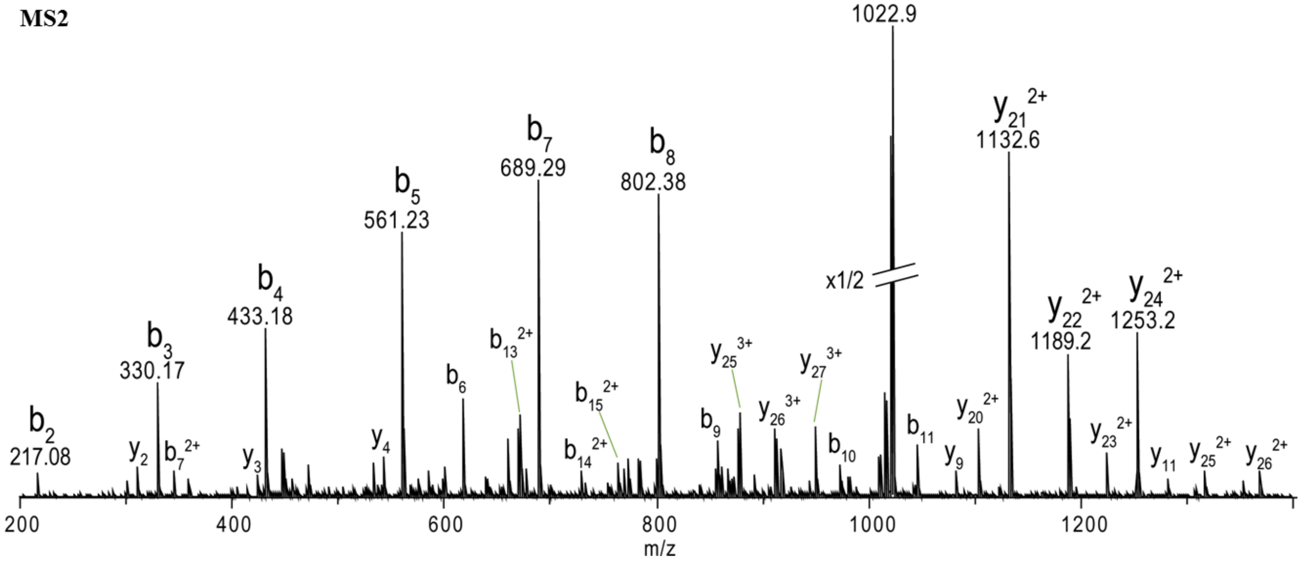

Supplementary Fig. 5 | Identification of µI-IIC115A by MS/MS annotation with LC-HR-MS/MS (qTOF). B) MS/MS annotation of peptide TDICQGALGDAWLLAAIASLTLNDTLLHR. (m/z = 1022.5, 3 charge).

A.

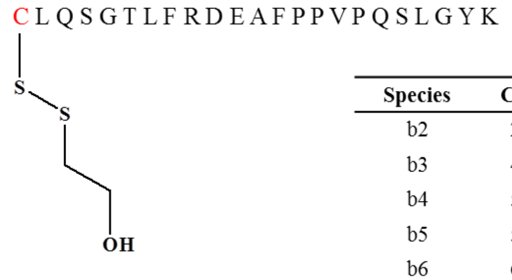

Species	Cal. mass	Obs. mass	Error(Da)	Species	Cal. mass	Obs. mass	Error(Da)
b2	293.099	293.099	0.000	b20^{+2}	1132.05	1132.05	0.00
b3	421.157	421.156	0.001	b22^{+2}	1242.09	1242.10	-0.01
b4	508.189	508.189	0.000	y3	367.198	367.197	0.001
b5	565.211	565.211	0.000	y4	480.282	480.280	0.002
b6	666.259	666.260	-0.001	y5	567.314	567.313	0.001
b7	779.343	779.337	0.004	y7	792.425	792.425	0.000
b8	926.411	926.408	0.003	y7^{+2}	396.716	396.715	0.001
b9	1082.51	1082.52	-0.01	y8	891.493	891.491	0.002
b10^{+2}	599.273	599.274	-0.001	y8^{+2}	446.250	446.251	-0.001
b11	1326.58	1326.58	0.00	y9	988.546	988.544	0.002
b11^{+2}	663.795	663.793	0.002	y10	1085.60	1085.60	0.00
b12	1397.62	1397.61	0.01	y10^{+2}	543.303	543.303	0.000
b12^{+2}	699.313	699.316	-0.003	y11	1232.67	1232.67	0.00
b12-H_2O^{+2}	690.308	690.308	0.000	y11^{+2}	616.837	616.838	-0.001
b13	1544.69	1544.68	0.01	y12	1303.71	1303.71	0.00
b13^{+2}	772.847	772.848	-0.001	y12^{+2}	652.356	652.360	-0.004
b13-H_2O	1526.68	1526.68	0.00	y15^{+2}	852.441	852.437	0.004
b13-H_2O^{+2}	763.842	763.841	0.001	y16^{+2}	925.975	925.979	-0.004
b14^{+2}	821.374	821.376	-0.002	y18^{+2}	1033.04	1033.04	0.01
b16^{+2}	919.434	919.433	0.001	y19^{+2}	1061.55	1061.56	-0.01
b16-H_2O^{+2}	910.429	910.425	0.004	y20^{+2}	1105.07	1105.07	0.00
b18^{+2}	1031.99	1031.98	0.01	y21^{+2}	1169.10	1169.10	0.00
b19^{+2}	1075.51	1075.50	0.01	y21^{+3}	779.734	779.730	0.004

MS1

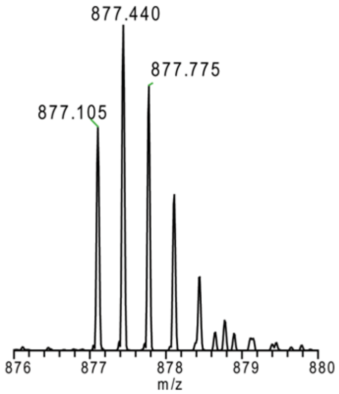

MS2

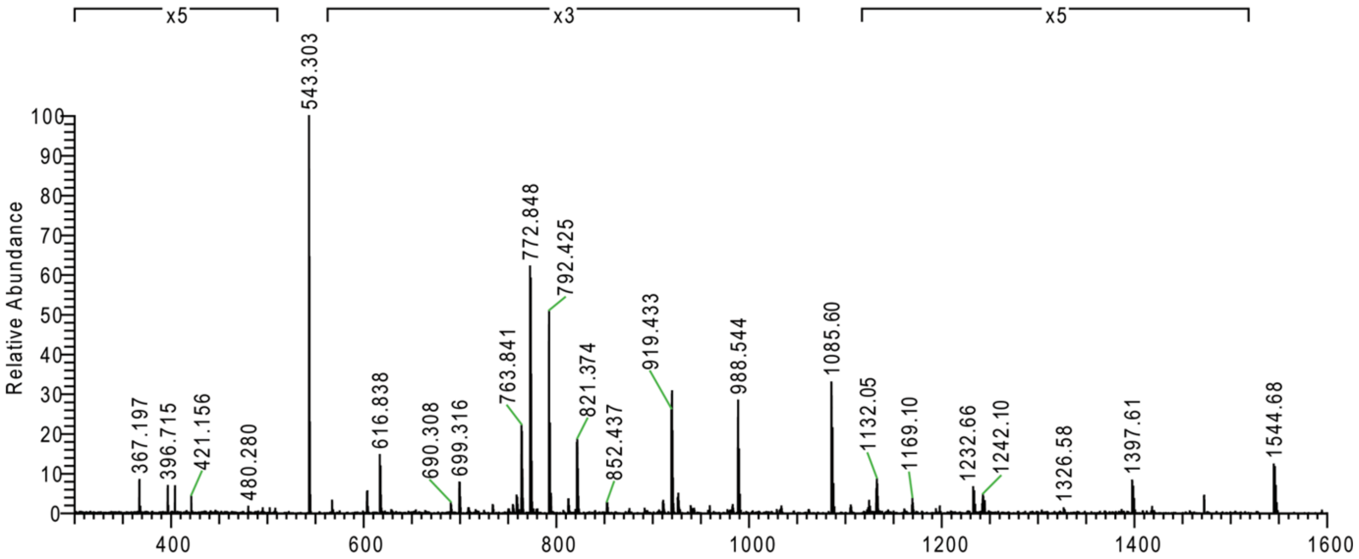

Supplementary Fig. 6 | MS/MS annotation of β-ME modified peptides in µI-II by FT-ICR-MS. A) A β-ME modification (+75.9983 Da) occurs at Cys49 of the peptide CLQSGTLFRDEAFPPVPQSLGYK.

B.

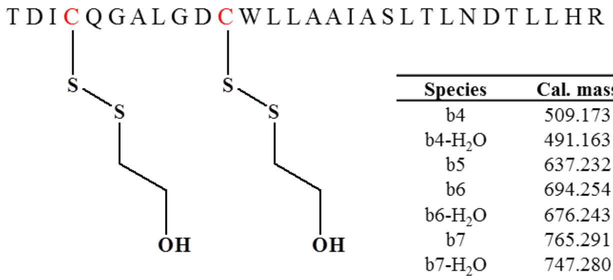

MS1

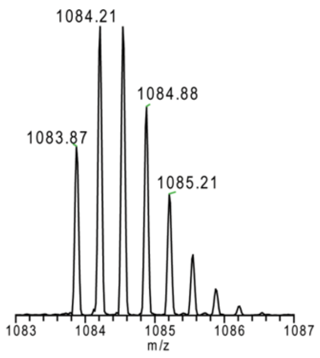

Species	Cal. mass	Obs. mass	Error(Da)	Species	Cal. mass	Obs. mass	Error(Da)
b4	509.173	509.172	0.001	y11	1282.71	1282.71	0.00
b4-H_2O	491.163	491.162	0.001	y12	1353.75	1353.75	0.00
b5	637.232	637.230	0.002	y12^{+2}	677.378	677.377	0.001
b6	694.254	694.252	0.002	y13	1466.83	1466.84	-0.01
b6-H_2O	676.243	676.242	0.001	y14	1537.87	1537.88	-0.01
b7	765.291	765.290	0.001	y14^{+2}	769.439	769.437	0.002
b7-H_2O	747.280	747.281	-0.001	y15	1608.91	1608.92	-0.01
b8	878.375	878.377	-0.002	y15^{+2}	804.957	804.958	-0.001
b8-H_2O	860.364	860.368	-0.004	y16^{+2}	861.499	861.502	-0.003
b9	935.396	935.402	-0.006	y17^{+2}	918.041	918.042	0.001
b9-H_2O	917.386	917.386	0.000	y18^{+2}	1011.08	1011.08	0.00
b10	1050.42	1050.43	-0.01	y19^{+2}	1100.59	1100.58	0.01
b10-H_2O	1032.41	1032.42	-0.01	y20^{+2}	1158.10	1158.10	0.00
b11	1229.43	1229.43	0.00	y20-H_2O^{+2}	1149.09	1149.10	-0.01
b12	1415.51	1415.51	0.00	y21^{+2}	1186.61	1186.61	0.00
b13	1528.59	1528.61	-0.02	y21-H_2O^{+2}	1177.60	1177.61	-0.01
b13-H_2O^{+3}	504.199	504.195	0.004	y22^{+2}	1243.15	1243.16	-0.01
b15	1712.72	1712.74	-0.02	y23^{+2}	1278.67	1278.67	0.00
b16^{+2}	892.380	892.383	-0.003	y24-H_2O^{+2}	1298.18	1298.17	0.01
b17	1896.84	1896.84	0.00	y25^{+2}	1371.21	1371.22	-0.01
y3	425.262	425.261	0.001	y25^{+2}	914.475	914.477	-0.002
y4	538.346	538.347	-0.001	y25-H_2O^{+2}	1362.20	1362.21	-0.01
y5	639.394	639.395	-0.001	y25-H_2O^{+2}	908.472	908.473	-0.001
y6	754.421	754.425	-0.004	y26^{+2}	1460.71	1460.71	0.00
y6-H_2O	736.410	736.410	0.000	y26^{+3}	974.145	974.143	0.002
y7	868.464	868.468	-0.004	y26-H_2O^{+2}	1451.71	1451.71	0.00
y8	981.548	981.545	0.003	y26-H_2O^{+3}	968.141	968.148	-0.007
y9^{+2}	541.801	541.801	0.000	y27^{+2}	1517.26	1517.24	0.02
y9-H_2O	1064.59	1064.58	0.01	y27^{+3}	1011.84	1011.84	0.00
y10	1195.68	1195.68	0.00	y27-H_2O^{+3}	1005.84	1005.84	0.00

MS2

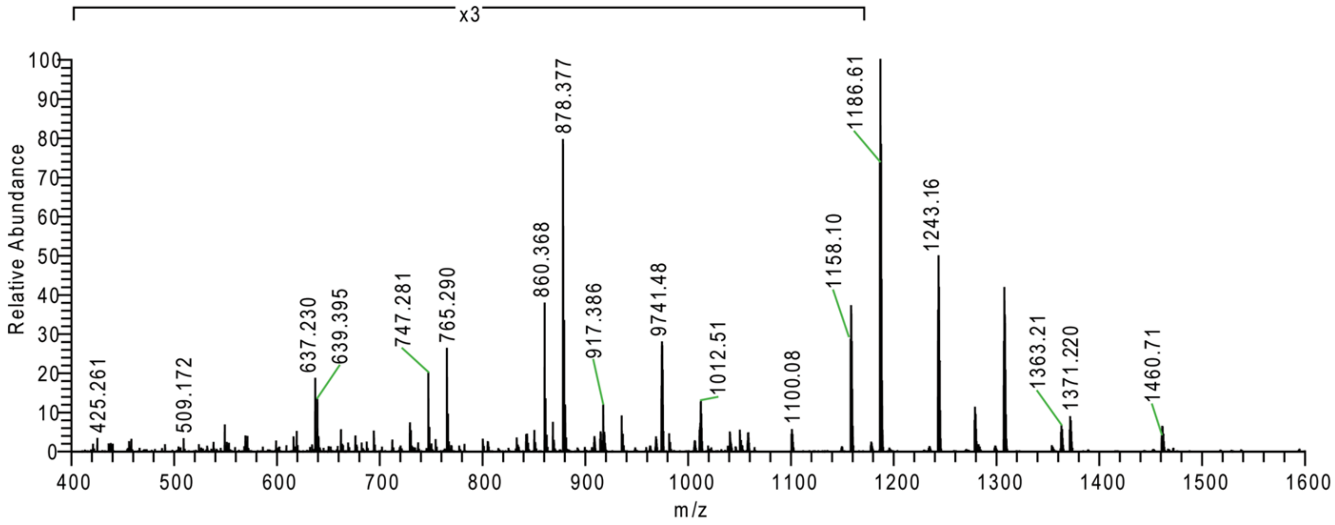

Supplementary Fig. 6 | MS/MS annotation of β-ME modified peptides in µI-II by FT-ICR-MS. B) β-ME modifications (+75.9983 Da) occur at Cys108 and Cys115 of the peptide TDICQGALGDCWLLAAIASLTLNDTLLHR.

A.

μI-II

Calculated exact mass: 42467.9 Da, Observed exact mass: 42467.9 Da, Error: 0.0 Da

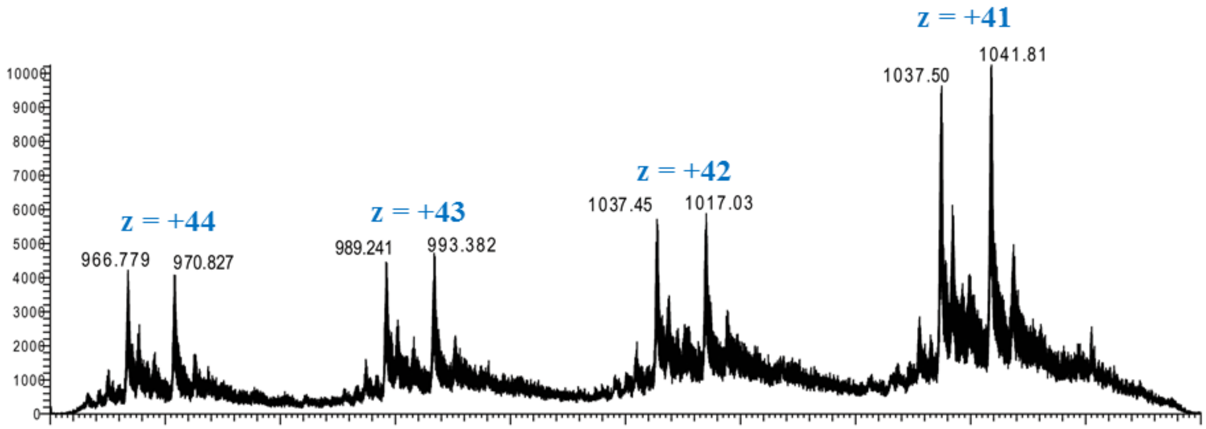

μI-II + 1 (shifted by 881.5 Da)

Calculated exact mass: 43349.4 Da, Observed exact mass: 43349.4 Da, Error: 0.0 Da

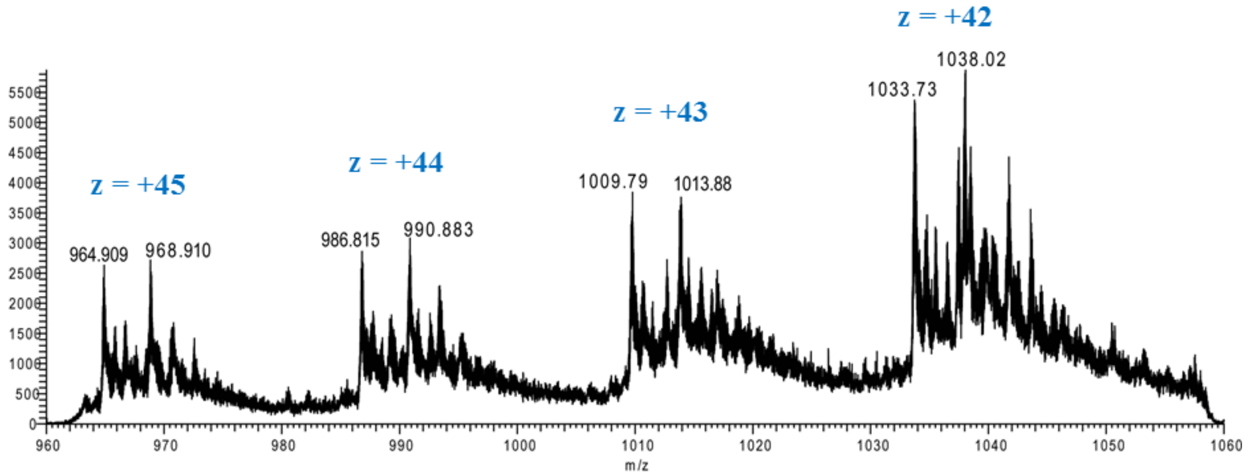

Supplementary Fig. 7 | FT-MS data comparison of μI-II and μI-IIC115A samples. A) μI-II and μI-II + **1** sample. A shift of 881.5 was observed in μI-II + **1** sample. The calculated exact mass of μI-II includes 3 β-ME adducts (75.9983 Da each). The calculated exact mass of μI-II + **1** includes 2 β-ME adducts and the mass of **1** (957.5423 Da).

B.

μI-IIC115A

Calculated exact mass: 42359.9 Da, Observed exact mass: 42360.1 Da, Error: -0.2 Da

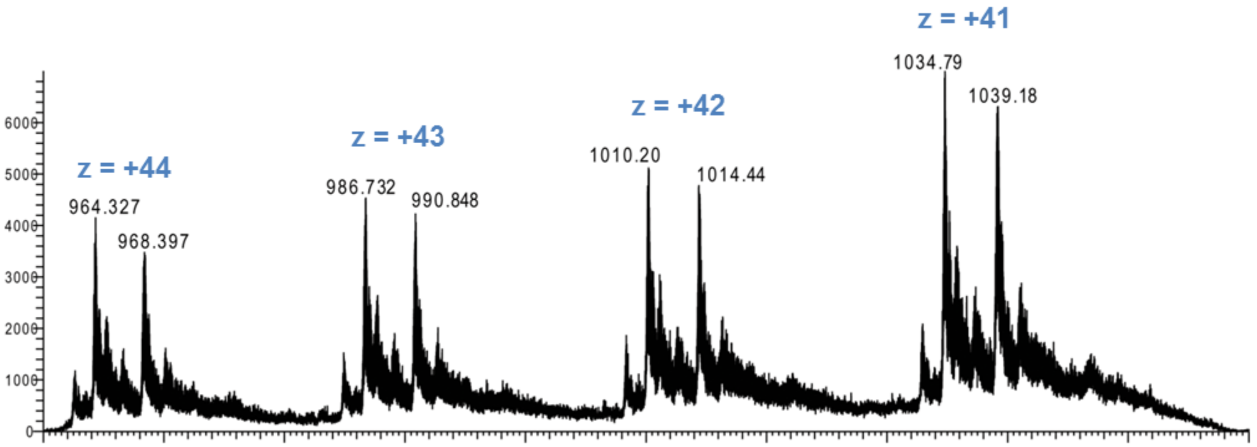

μI-IIC115A+ 1 (no shift)

Calculated exact mass: 42359.9 Da, Observed exact mass: 42360.1 Da, Error: -0.2 Da

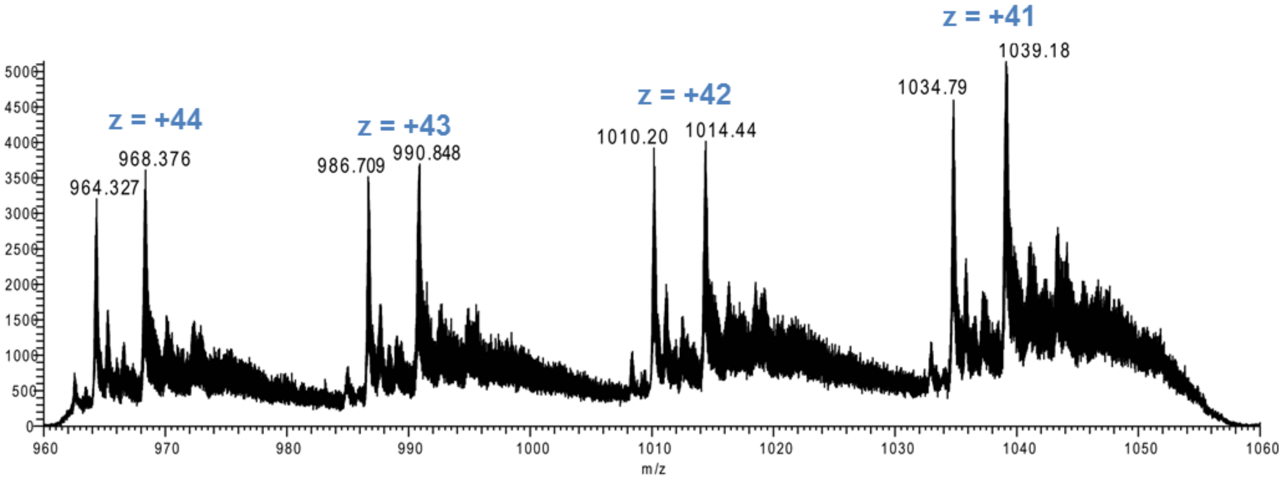

Supplementary Fig. 7 | FT-MS data comparison of μI-II and μI-IIC115A samples. B) μI-IIC115A and μI-IIC115A + **1** sample. No shift was observed in μI-IIC115A + **1** sample. The calculated exact masses of μI-IIC115A and μI-IIC115A + **1** both include 2 β-ME adducts (75.9983 Da each).

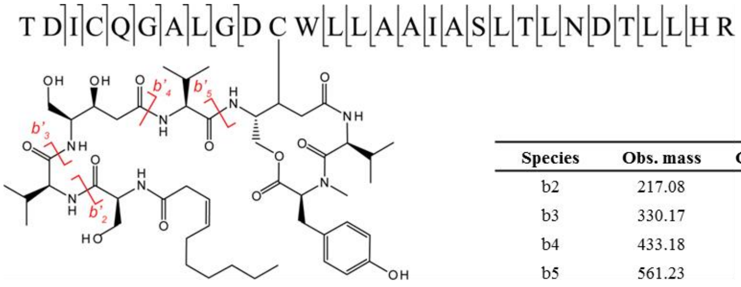

MS1

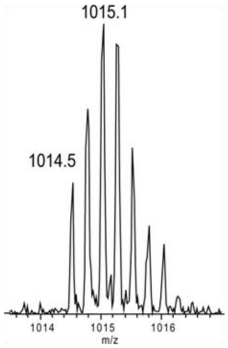

Species	Obs. mass	Cal. mass	Error(Da)	Species	Obs. mass	Cal. mass	Error(Da)
b2	217.08	217.08	0.00	y11	1282.7	1282.7	0.0
b3	330.17	330.17	0.00	y11^{+2}	641.86	641.85	0.01
b4	433.18	433.17	0.01	y12^{+2}	677.38	677.38	0.00
b5	561.23	561.24	-0.01	y13^{+2}	733.92	733.92	0.00
b6	618.26	618.26	0.00	y14^{+2}	769.44	769.44	0.00
b7	689.29	689.29	0.00	y15^{+2}	804.96	804.96	0.00
b8	802.38	802.38	0.00	y16^{+2}	861.50	861.50	0.00
b9	859.40	859.40	0.00	y17^{+2}	918.04	918.04	0.00
y2	312.18	312.18	0.00	y21^{+3}	1085.3	1085.3	0.0
y3	425.26	425.26	0.00	y22^{+3}	1123.0	1123.0	0.0
y4	538.35	538.34	0.01	b'2	240.16	240.16	0.00
y5	639.39	639.39	0.00	b'3	339.23	339.23	0.00
y5^{+2}	320.20	320.21	-0.01	b'4	470.29	470.29	0.00
y6	754.42	754.42	0.00	b'5	569.35	569.35	0.00
y6^{+2}	377.71	377.70	0.01	[M+4H+]-b'3 $^{+4}$	929.98	929.97	0.01
y7	868.46	868.47	-0.01	[M+4H+]-b'2 $^{+4}$	954.75	954.74	0.01
y8	981.55	981.54	0.01	[M+3H+]-b'5 $^{+3}$	1162.9	1162.9	0.0
y9	1082.6	1082.6	0.0	[M+3H+]-b'3 $^{+3}$	1239.6	1239.7	-0.1
y10	1195.7	1195.7	0.0				

MS2

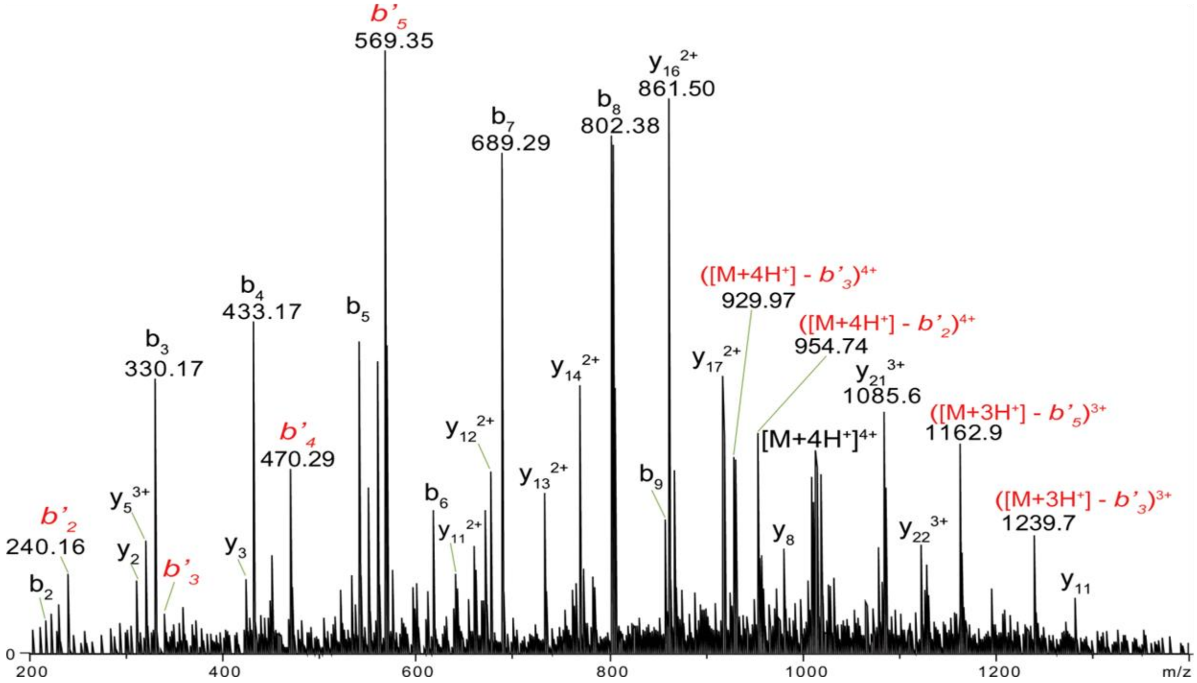

Supplementary Fig. 8 | MS/MS annotation of **1**-modified peptide by Q-TOF (m/z= 1014.5, +4)**.** B ions of **1** were labeled by b' and red color.

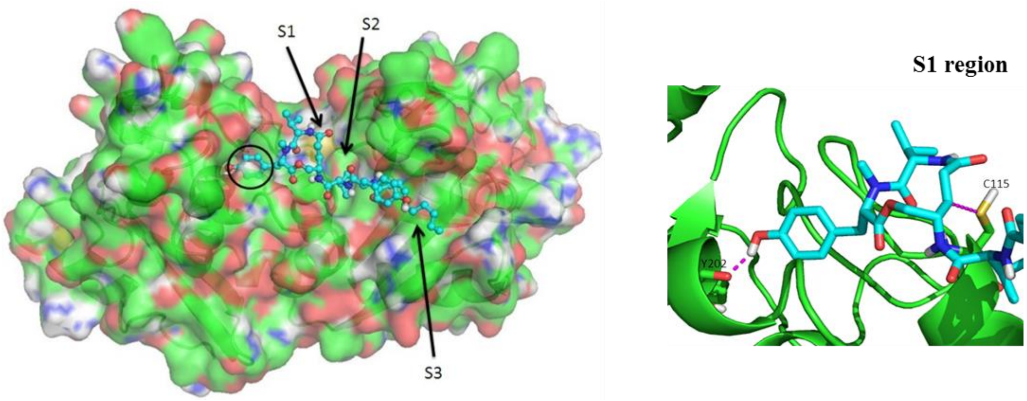

Supplementary Fig. 9 | Representative pose of **4 (**thalassospiramide C) in the calpain model. The lowest energy binding conformation of **4** shows the best pose to S1, S2 and S3 position of calpain in the model. The double bond in the ring has a short distance to Cys115 and the phenol group forms a hydrogen bond with Y202 (the enlarged view of S1 region shown on the right).

ESI-TOF-HRMS

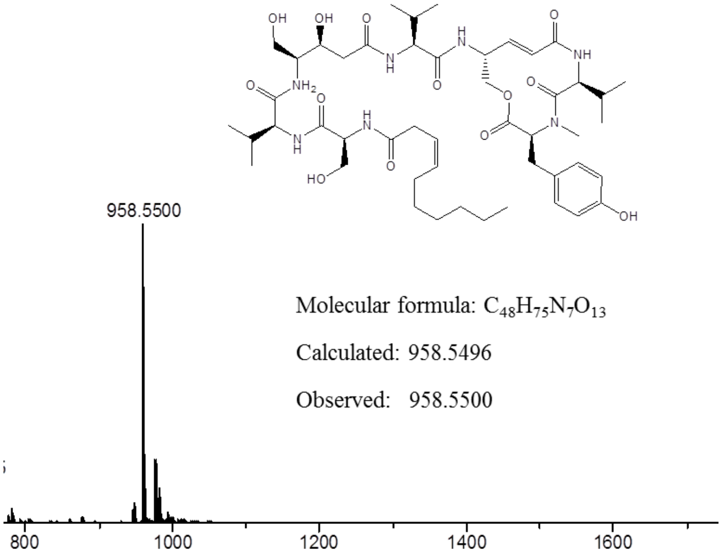

H[1] NMR

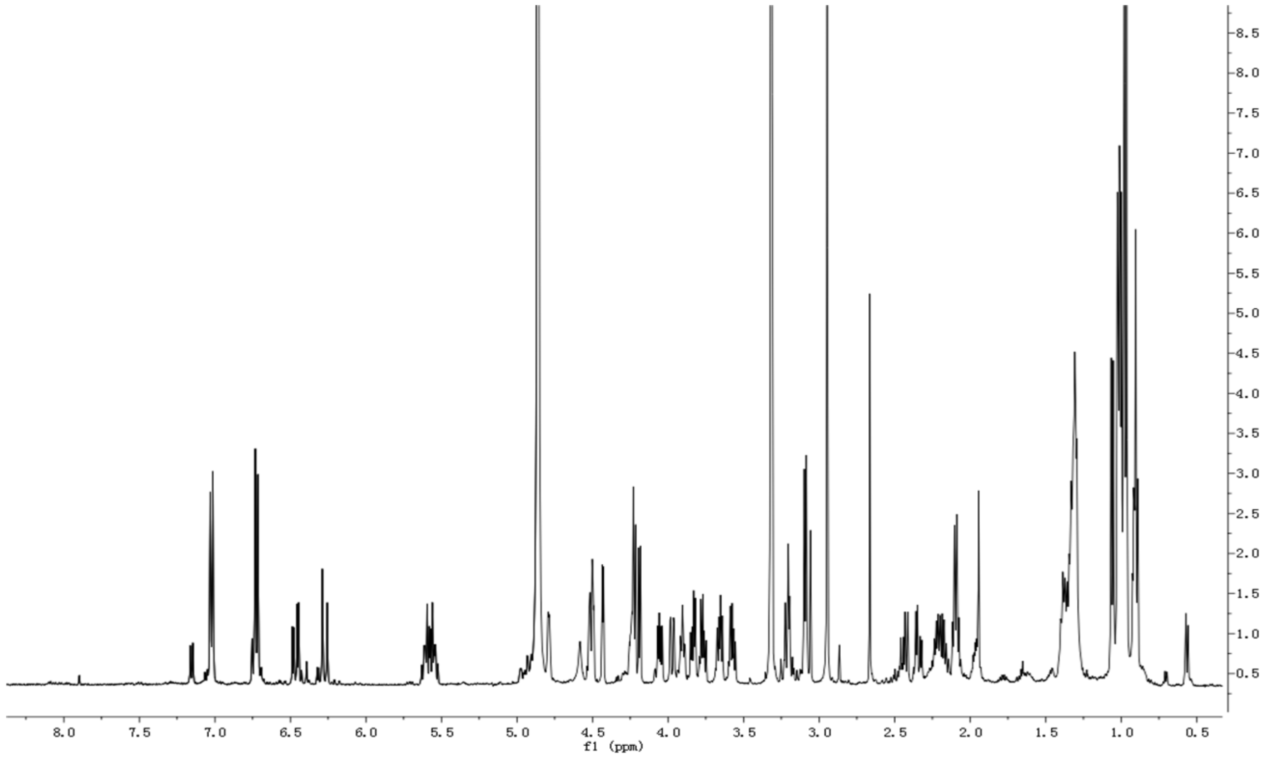

Supplementary Fig. 10 | Characterization of **1** by HRMS and ^{1}H NMR (in methanol-d_4).

MS¹ (ESI-TOF-HRMS)

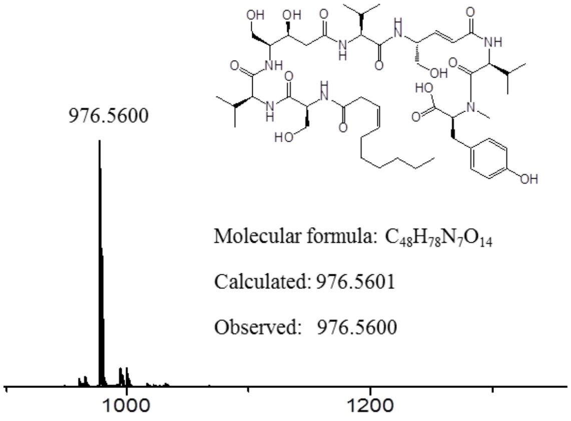

MS³ (781.39 m/z)

Species	Cal. mass	Obs. mass	Error (Da)
b4	470.29	470.42	-0.13
b5	569.36	569.30	0.06
b6	682.40	682.43	-0.03
y3	312.18	312.23	-0.05
y4	443.24	443.22	0.02
y5	542.31	542.28	0.03
y6	629.34	629.44	-0.10

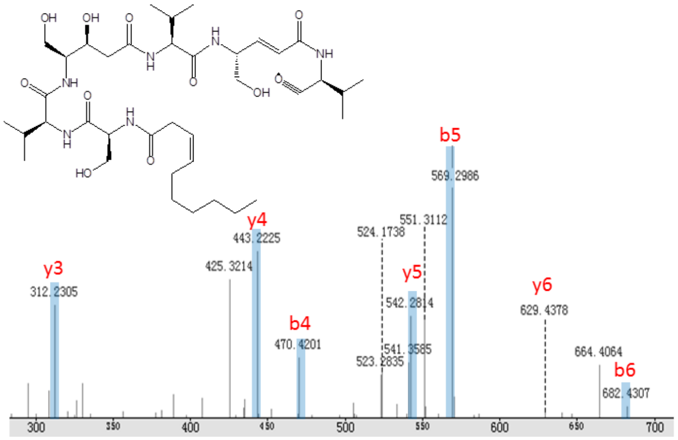

MS² (976.56 m/z)

Species	Cal. mass	Obs. mass	Error (Da)
b3(-18)	321.23	321.18	0.05
b4	470.29	470.21	0.08
b5	569.36	569.22	0.14
b6	682.40	682.37	0.03
b7	781.47	781.38	0.09
y3	408.20	408.20	0.00
y4	507.27	507.30	-0.03
y5	638.32	638.35	-0.03

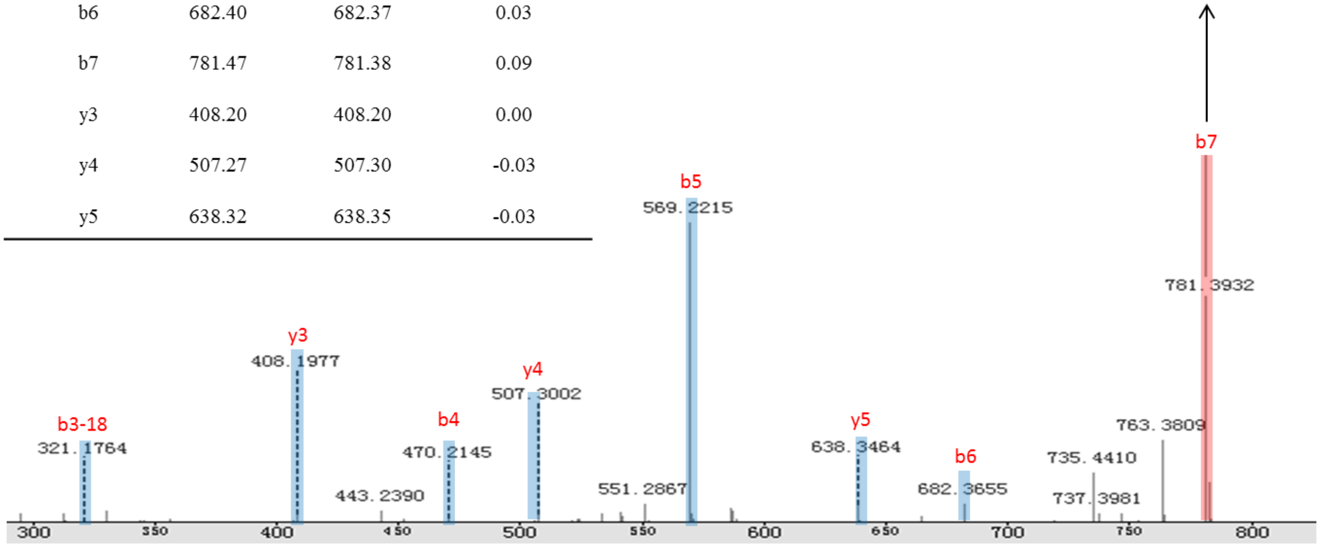

Supplementary Fig. 11 | Characterization of **2** by HRMS and MSⁿ.

ESI-TOF-MS

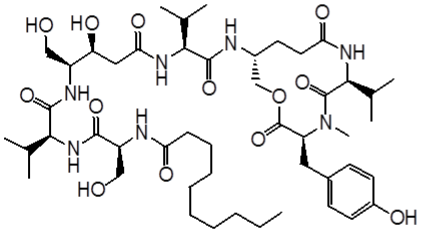

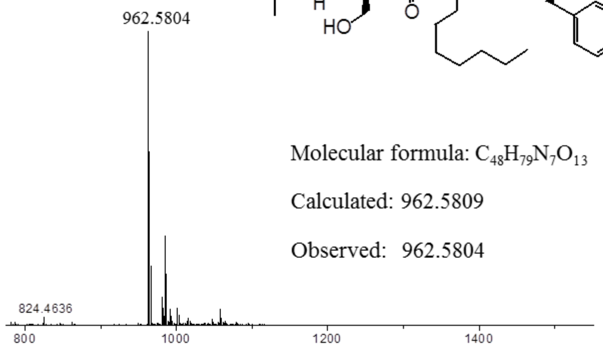

Molecular formula: $C_{48}H_{79}N_7O_{13}$

Calculated: 962.5809

Observed: 962.5804

Species	Cal. mass	Obs. mass	Error (Da)
b2	242.18	242.27	-0.09
b3	341.24	341.24	0.00
b4	472.28	472.30	-0.02
b5	571.37	571.35	0.02
y2	392.20	392.21	-0.01
y3	491.27	491.27	0.00
y4	622.33	622.33	0.00
y5	721.40	721.40	0.00

MS2 – 962.58 m/z

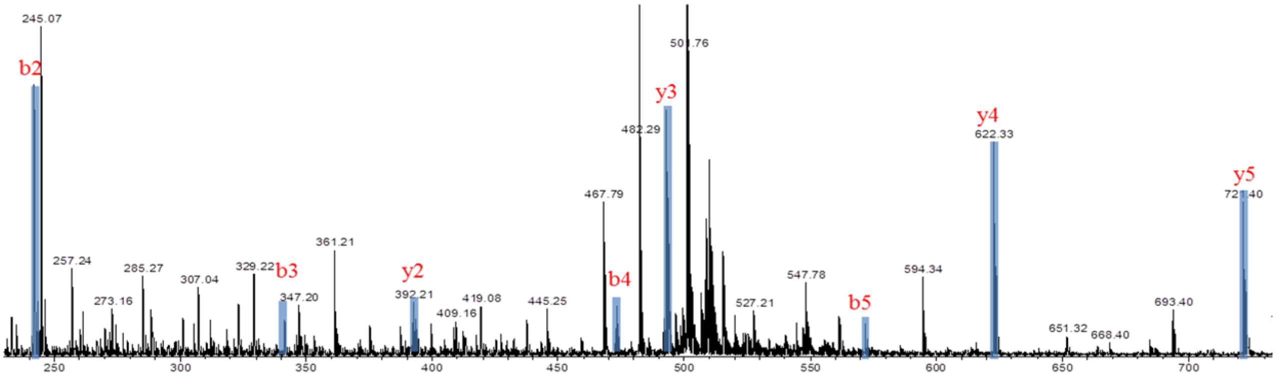

Supplementary Fig. 12 | Characterization of **3** by HRMS and MS^n.

ESI-TOF-HRMS

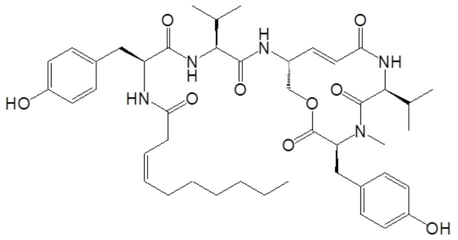

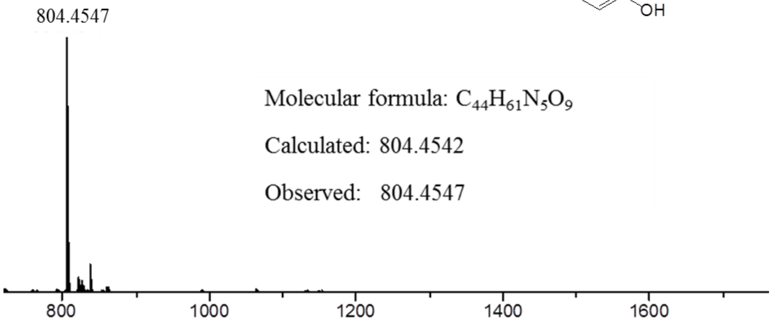

H[1] NMR

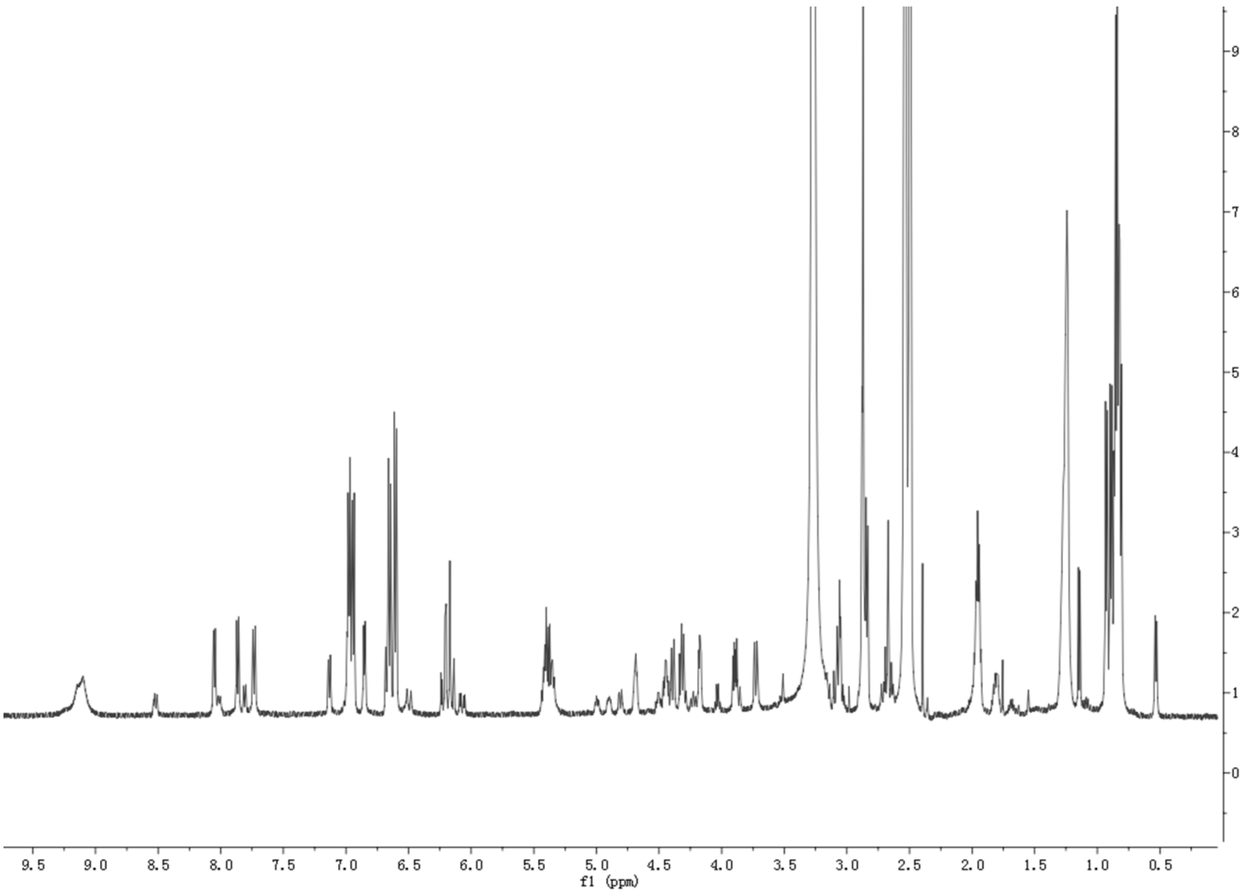

Supplementary Fig. 13 | Characterization of **4** by HRMS and ^{1}H NMR (in methanol-d_4).

Supplementary Tables

Supplementary Table 1: Docking results for the representative thalassospiramides.

Compound	Minimum warhead distance (Å)	Number of docking poses with distance[a] <5Å	Minimum AutoDock score (kcal/mol)	IC_{50} (nM)
Thalassospiramide A	4.29	2	-6.2	57
Thalassospiramide A1	3.98	5	-7.3	42
Thalassospiramide A5	4.95	3	-7	22
Thalassospiramide D	4.64	3	-7.4	21
Thalassospiramide D1	3.7	8	-6.8	35
Thalassospiramide B	3.98	10	-6.8	29
Thalassospiramide C	3.39	20	-8.1	3
Thalassospiramide E1	3.60	6	-7.6	21

[a]: the distance between warhead carbon and active site sulfur.
